# Shaping the cognitive reserve: the role of lifelong enrichment and education in the Alzheimer’s disease continuum

**DOI:** 10.3389/fnagi.2026.1736047

**Published:** 2026-05-14

**Authors:** Laura Serra, Sabrina Bonarota, Carlotta Di Domenico, Giulia Caruso, Martina Rizzuti, Sofia Sperati, Federico Maria Tamigi, Francesco Di Lorenzo, Francesco Ricci, Marco Laudadio, Giacomo Koch, Federico Giove, Carlo Caltagirone, Marco Bozzali

**Affiliations:** 1 Department of Human Sciences, Università degli Studi Guglielmo Marconi, Rome, Italy; 2 Neuroimaging Laboratory, Fondazione Santa Lucia IRCCS, Rome, Italy; 3 Department of Psychology, Sapienza University of Rome, Rome, Italy; 4 Experimental and Behavioral Neurophysiology Laboratory, Fondazione Santa Lucia, Rome, Italy; 5 Experimental Neuropsychophysiology Laboratory, Fondazione Santa Lucia IRCCS, Roma, Italy; 6 Neurology 2 Unit, Department of Neurosciences “Rita Levi Montalcini”, University of Turin, Torino, Italy; 7 A.O.U. Città della Salute e Della Scienza di Torino, Turin, Italy; 8 Department of Neuroscience and Rehabilitation University of Ferrara, Ferrara, Italy; 9 Museo Storico della Fisica e Centro Studi e Ricerche Enrico Fermi, Rome, Italy; 10 Scientific Direction, Fondazione Santa Lucia IRCCS, Rome, Italy

**Keywords:** Alzheimer’s disease, cognitive activities, cognitive reserve, leisure activities, mild cognitive impairment, physical activities, social activities, subjective cognitive decline

## Abstract

**Background:**

Leisure activities (LAs) are recognized as major contributors to cognitive reserve (CR), potentially mitigating age-related cognitive decline and dementia progression. However, the specific associations between lifelong engagement in different types of LAs, education (as a proxy of CR), and cognitive functioning along the Alzheimer’s disease (AD) continuum remain poorly defined. This study aimed to investigate the association between education and LAs in relation to current cognitive performance in healthy subjects (HS), individuals with subjective cognitive decline (SCD), amnestic mild cognitive impairment (a-MCI), and AD patients.

**Methods:**

Two hundred eighty-six participants (82 AD, 98 a-MCI, 39 SCD, 67 HS) underwent comprehensive neuropsychological testing and completed a validated questionnaire assessing the frequency of cognitive (C1–C8), social (S1–S5), and physical (P1–P5) activities during youth, middle, and late adulthood. Associations among education, LAs, and cognitive outcomes were analyzed using Spearman’s correlations and moderation analyses, while between-group differences were explored with Kruskal–Wallis ANOVAs.

**Results:**

Education showed significant correlations with most LA domains across all groups. Distinct activity-cognition patterns emerged along the disease continuum. In HS, participation in individual sports during youth and midlife correlated with global cognitive efficiency and memory. In SCD individuals, pet care during midlife was associated with memory, whereas in a-MCI patients, attending lectures and sewing/knitting were related to current cognitive efficiency. No significant associations were observed in AD patients. Moderation analyses revealed that education was significantly associated with the relationship between LA engagement and cognitive outcomes in HS, SCD, and a-MCI, but not in AD. Kruskal-Wallis analyses indicated reduced engagement in cognitive and social activities across disease stages compared with HS, particularly during youth and midlife.

**Discussion:**

Differences in lifelong LA engagement were evident across diagnostic groups, and education was associated with variations in cognition-activity relationships. These findings suggest that the role of CR through leisure engagement may vary across disease stages.

**Conclusion:**

Education-related cognitive reserve was linked to the relationship between lifelong leisure activities and cognitive performance in healthy individuals and early-stage AD conditions, but not in advanced AD, highlighting the potential role of early-life cognitive, social, and physical enrichment in supporting preserved cognitive function.

## 1 Introduction

The concept of reserve mechanisms and their neuroprotective role against cognitive decline in neurodegenerative conditions has gained substantial empirical support over recent decades (Stern et al., 2020). These mechanisms encompass brain reserve, cognitive reserve, and neural reserve, each reflecting distinct yet interrelated properties of cerebral architecture and function. Such properties include neuronal density, dendritic arborization, brain volumes, and white matter connectivity patterns, collectively contributing to enhanced cognitive processing efficiency (Cabeza et al., 2018; Stern et al., 2020; Stern et al., 2023; Serra et al., 2022a).

Longitudinal studies involving patients with amnestic Mild Cognitive Impairment (a-MCI), a preclinical stage of Alzheimer’s Disease (AD), have consistently demonstrated that patients with higher reserve levels have been reported to show a later clinical manifestation of symptoms compared to those with lower reserves (Serra et al., 2015). This association has been interpreted as potentially reflecting neural adaptation mechanisms, enabling maintained cognitive performance despite the accumulation of AD pathological changes, including beta-amyloid deposits and tau pathology. The underlying mechanisms involve enhanced neural connectivity and compensatory recruitment of alternative neural circuits, effectively counterbalancing neuronal loss (Bozzali et al., 2015; Serra et al., 2017a; Franzmeier et al., 2024).

Neuroimaging studies across different AD clinical stages have suggested that individuals with higher educational attainment or exposure to cognitively stimulating experiences tend to show greater structural and functional brain integrity, which may support preserved cognitive functions (Serra et al., 2011; Bozzali et al., 2015; Serra et al., 2017a; Zhu et al., 2021; Zhukovsky et al., 2023). For instance, larger gray matter volumes and more efficient neural connectivity patterns have been associated with resilience against cognitive deterioration (Serra et al., 2011; Serra et al., 2019; Serra et al., 2017b; Serra et al., 2021; van Loenhoud et al., 2022). In the present study, however, brain resilience is considered primarily as a conceptual framework derived from the existing literature, referring to the ability to maintain cognitive functioning despite neuropathological burden. As no structural or functional neuroimaging measures were included, this construct is explored indirectly through behavioral and cognitive indicators, such as education, leisure activities, and cognitive performance.

Education is considered one of the most important factors influencing brain resilience and promoting cognitive reserve (Serra et al., 2018; Stern, 2012; Dufouil et al., 2003). Studies have shown that higher educational levels are associated with an enrichment of neocortical synapses, leading to increased structural and functional connectivity (Serra et al., 2018; Katzman, 1993; Serra et al., 2017a). Furthermore, individuals with higher levels of education are more likely to engage in cognitively stimulating activities throughout their lives (Vance and Crowe, 2006), which have been associated with later clinical manifestation (Katzman, 1993).

The relationship between education and socialization has also been demonstrated, suggesting that higher education facilitates more efficient learning strategies and responses to cognitive requirements (Gilleard, 1997). In addition, the protective role of leisure activities (LAs) in promoting the reserves and cognitive efficiency has been extensively documented (Serra et al., 2015; Gelfo et al., 2024; Serra et al., 2022b; Park et al., 2019; Fancourt et al., 2021; Cai et al., 2023; Pappalettera et al., 2024). Both human and animal studies support the view that cognitive, social, and physical activities are linked to enhanced brain function and cognitive performance during normal and pathological aging (Serra et al., 2015; Gelfo et al., 2024; Serra et al., 2022b; Stern and Munn, 2010; Verghese et al., 2003; Wang et al., 2012; Livingston et al., 2020). Recent findings indicate differential contributions of these activities across various AD stages (Serra et al., 2024). Specifically, the combined engagement in cognitive, social, and physical activities is straightly associated with efficient cognitive functioning in cognitively healthy elderly individuals and in those with subjective cognitive decline (SCD). In contrast, cognitive recreational activities seem to play a predominant role in supporting cognition in patients at the stage of mild cognitive impairment (MCI), while social activities emerge as the primary predictor of cognitive performance at the stage of dementia.

While there is scientific consensus regarding the classification of activities as cognitive (e.g., reading books, newspapers, writing narratives, playing instruments, drawing), social (e.g., meeting friends or relatives, volunteering, playing cards or board games), or physical (e.g., cycling, skating, skiing, boating, running, dancing, practicing yoga, team sports) (Tinsley and Eldredge, 1995; Scarmeas et al., 2001; Verghese et al., 2003; Scarmeas and Stern, 2003; Verghese et al., 2006; Wang et al., 2006; Wilson et al., 2007; Stern and Konno, 2009; Stern and Munn, 2010; Stern, 2012; Fancourt et al., 2021; Song et al., 2022; Armstrong et al., 2022; Akbayrak et al., 2024), the precise relationship between cognitive reserve, different types of leisure activities, and current cognitive status across the AD continuum remains underexplored.

This gap of knowledge is particularly important given the potential implications for preventive interventions and therapeutic strategies.

The present study aimed to explore the relationship of different types of leisure activities (LAs) along the Alzheimer’s Disease continuum and to investigate the role of cognitive reserve, operationalized as educational level, in moderating the association between lifelong engagement in these activities and current cognitive performance. Building on well-established previous evidence linking cognitive reserve (CR) proxies to brain reserve (BR) using structural and functional neuroimaging (Medaglia et al., 2017; Giovacchini et al., 2019; Vockert et al., 2024; Guo et al., 2025), this study specifically focused on behavioral measures, evaluating whether a brief, clinically applicable questionnaire could effectively capture the relationship between education, recreational activity engagement, and cognitive outcomes across the disease spectrum. By adopting this approach, we provide a practical tool for assessing CR-related associations in clinical settings, complementing existing neuroimaging-based findings.

## 2 Methods

Two hundred and 86 participants, including 82 patients with AD, 98 with amnestic MCI (a-MCI), 39 individuals with SCD, and 67 healthy subjects (HS), were consecutively recruited at the Memory Clinic of Fondazione Santa Lucia IRCCS (Rome, Italy). All subjects underwent a comprehensive neuropsychological screening and a questionnaire devoted to the assessment of leisure activities (Serra et al., 2015; Serra et al., 2024). Moreover, all participants underwent an MRI scan at 3 T that allowed semiquantitative assessment of medial temporal lobe atrophy.

The diagnosis of probable AD met the latest research criteria (Jack et al., 2024), while the diagnosis of a-MCI met the current diagnostic criteria (Albert et al., 2011). A-MCI patients were all single-domain. By definition, they had not to respond to the diagnostic criteria for major cognitive disorders (Albert et al., 2011), their Clinical Dementia Rating (Hughes et al., 1982) score had not to exceed 0.5, and their MMSE score (Folstein et al., 1975; Magni et al., 1996) had to fall within the cut-off of normality (>23.8). SCD individuals were required to meet the following inclusion criteria: the presence of subjective memory complaint in daily life; no evidence of cognitive deficits in memory or in other domains on formal neuropsychological testing; the absence of any other clinical condition accounting for their cognitive symptoms. HS participants had not to report any subjective cognitive complaint in daily living, they had to score normally in all cognitive domains on formal neuropsychological testing, and they had not to show any significant atrophy in their medial temporal lobes. Participants from any considered group with a Hachinski score (Hachinski et al., 1975) higher than 4 were excluded. Major systemic, psychiatric, and other neurological illnesses were also carefully investigated and excluded in all participants. Finally, all subjects were required to be right-handed, as assessed by the Edinburgh Handedness Inventory (Büsch et al., 2010).

The study was approved by the Ethics Committee of Fondazione Santa Lucia IRCCS, and written informed consent was obtained from all participants and/or their legal guardians before study initiation. All procedures performed in this study are in accordance with the 1964 Helsinki declaration and its later amendments or comparable ethical standards.

### 2.1 Neuropsychological assessment

All participants underwent an extensive neuropsychological assessment covering all cognitive domains: (a) verbal episodic long-term memory: 15-Word List (Immediate and 15-min Delayed recall, and recognition) (Carlesimo et al., 1996); Short Story Test (Immediate and 20-min Delayed recall) (Carlesimo et al., 2002); (b) visuo-spatial long-term memory: Complex Rey’s Figure (Immediate and 20-min Delayed recall) (Carlesimo et al., 1996); (c) short-term and working memory: Digit span (forward and backward) and Corsi Block Tapping task (forward and backward) (Monaco et al., 2013); (d) executive functions: Phonological Word Fluency (Carlesimo et al., 1996) and Modified Card Sorting Test (Nocentini et al., 2002); (e) language: Naming objects subtest of the BADA (“Batteria per l’Analisi dei Deficit Afasici,” Italian for “Battery for the analysis of aphasic deficits”) (Miceli et al., 1991); (f) Reasoning: Raven’s Colored Progressive Matrices (Carlesimo et al., 1996); (g) constructional praxis: copy of simple drawings with and without landmarks (Carlesimo et al., 1996), and copy of Complex Rey’s Figure (Carlesimo et al., 2002); (h) general cognitive efficiency: Mini Mental State Examination (MMSE) (Folstein et al., 1975; Magni et al., 1996).

For the purposes of the current study, focussed on the cognitive reserve, neuropsychological scores were not adjusted for age and education, as previously reported (Serra et al., 2011).

### 2.2 Leisure activities investigation

All participants underwent the Global Enrichment-Index (GE-I) questionnaire, a tool specifically devoted to extensively assessing the activities that are more likely to impact on cognitive reserve. The questionnaire is administered directly to participants whenever possible. As previously described (Serra et al., 2015; Serra et al., 2024), the GE-I questionnaire consists of three different sections: (A) information about years and type of education (Educational Attainment EA section); (B) information about type of main occupations (Occupational Attainment, OE section), and (C) information about the type and frequency of leisure activities (Leisure Activities, LAs section) pursued during the entire lifespan. LAs section investigates cognitive, social and physical activities. The LAs section of the GE-I questionnaire evaluates participation in leisure activities through specific items focusing on habitual engagement rather than specific episodic events. Participants were required to report the frequency by which they have been engaged in all activities using a five-point Likert scale (5 = daily; 4 = several times a week; 3 = several times a month; 2 = several times a year; 1 = never). The LAs section includes 8 items for cognitive activities (C1 = Participating in lectures/conferences/discussion groups; C2 = Reading books, newspapers; C3 = Producing non-artistic writings; C4 = Producing artistic works; C5 = Going to the cinema, theater, museums, art galleries, etc.; C6 = Playing a musical instrument; C7 = Playing structured games; C8 = Participating in hobbies), 5 items for social activities (S1 = Volunteering; S2 = Going to restaurants, bars, or cafes with friends or relatives; S3 = Taking care of pets or farm animals; S4 = Receiving friends or relatives at home/Going to the homes of relatives/friends; S5 = Participating in group activities), and 5 items for physical activities (P1 = Playing team sports; P2 = Walking or hiking for exercise or enjoyment; P3 = Practicing individual sports; P4 = Cycling, skating, skiing, canoeing, running; P5 = Embroidery, sewing, knitting). Each activity was assessed across three life periods: youth (age range: 20–40 years), middle age (age range: 40–65 years) and older age (65 years or older). For the specific purpose of the present study, we included in the analyses only LAs section part of the GE-I questionnaire.

### 2.3 Education measurement

To quantify the “years of education” variable, we calculated the total duration of formal schooling successfully completed by participants, starting from the first year of primary school. Given that our cohort was educated in Italy approximately between the 1950s and 1980s, the calculation follows the Italian ministerial educational tracks of that period:

1. Primary School: 5 years;
2. Lower Secondary School: 3 years;
3. Upper Secondary School: 3–5 years, depending on the track (vocational, technical, or classical/scientific lyceum). Only the 5-year diplomas allowed full access to the University;
4. Higher Education: 4–6 years (depending on the degree type, e.g., Humanities vs. Medicine).
5. Post-Graduate Education: To accurately reflect the highest levels of cognitive reserve, additional years were summed for participants who completed:

 -Specialization Schools: Typically 4 or 5 years (mandatory for medical doctors and psychotherapists).
 -PhD Programs (Dottorato di Ricerca): Usually 3 to 4 years of advanced research training.

#### 2.3.1 Calculating “years of education”

Education was treated as a continuous variable. For example, a participant with a high school diploma was assigned 13 years (5 + 3 + 5), while a participant with a degree was assigned 17 or 18 years.

In our study, part-time or evening vocational courses were included in the total count only if they led to an officially recognized qualification equivalent to full-time schooling. The “years of education” were calculated based on the legal duration of the course (e.g., a 3-year vocational diploma was counted as 3 years, regardless of whether it was attended part-time), as this reflects the formal level of cognitive challenge and attainment reached by the participant.

### 2.4 MRI acquisition

All participants underwent brain MRI-3 T by using Allegra or Prisma scanner (Siemens, Medical solutions, Erlangen, Germany) including the following acquisitions: (a) dual-echo spin echo (DE-SE) (TR = 5,000 ms, TE = 20/100 ms); (b) fast-fluid attenuated inversion recovery (FLAIR) (TR = 8,170 ms, TE = 96 ms, TI = 2,100 ms); (c) 3D T1-weighted (TR = 7.92 ms, TE = 2.4 ms, TI = 210 ms, flip angle = 15°). For the dual-echo and FLAIR scans, 52 contiguous interleaved axial slices were acquired with a 2 mm slice thickness, with a 192 × 256 matrix over a 256 mm × 256 mm field of view, covering the whole brain. The T1-weighted volumes were acquired in a single slab, with a sagittal orientation and 224 × 256 matrix size over a 256 × 256 mm2 field of view, with an effective slice thickness of a 1 mm.

MRI scans were visually reviewed by expert neuroradiologists to exclude macroscopic brain abnormalities.

#### 2.4.1 Medial temporal lobe atrophy

The Medial Temporal lobe Atrophy scale (MTA) (Scheltens et al., 1995) was employed on T1-weighted volumetric images to assess the severity of atrophy in each subject. This scale provides a rating score from 0 to 4, with scores > 1.5 (Pereira et al., 2014) indicating significant atrophy. For each subject, we averaged the scores obtained in the right and left hemisphere to obtain a single measure of medial-temporal lobe atrophy.

### 2.5 Statistical analyses

#### 2.5.1 Demographical features, clinical and neuropsychological features

SPSS-25.0^1^ was used for statistical analyses. A series of one-way ANOVAs were conducted to assess between-group differences in age, years of formal education, MTA scores, MMSE scores and neuropsychological test scores. Chi-square analysis was used to examine sex distribution.

Kruskal-Wallis’s ANOVAs were performed to assess between-group differences in the occurrence of different type of LAs, with the Dwass-Steel-Critchlow-Flinger test used for *post-hoc* comparisons. Statistical significance was set at *p* = 0.05.

Spearman’s coefficients were computed to assess associations between education, LAs items (each activity was separately considered) and cognition. In order to reduce the I-type risk error, correlation analyses were limited to measures of general cognition (i.e., MMSE score) and verbal episodic memory (i.e., RAVLT immediate recall: RAVLT-IR, and delayed recall: RAVLT-DR). Bonferroni’s correction was applied with ⍺ set at 0.05/18 (*p* = 0.003).

Finally, moderation analyses were conducted to investigate whether the relationship between LAs and the cognitive measure varied as a function of education. The analyses were performed using the PROCESS macro for SPSS implemented in IBM SPSS Statistics, following the conditional process modeling framework described by Andrew F. Hayes. Specifically, moderation effects were tested using ordinary least squares regression models corresponding to PROCESS Model 1 (a single model was estimated) (Hayes, 2018), In these analyses, each LA variable was entered as an independent predictor (X), years of education as the moderator (M), and the cognitive measures (MMSE, RAVLT-IR, and RAVLT-DR scores) as dependent variables (Y). Continuous predictors were mean-centered before analysis to reduce multicollinearity (as assessed by Variance Inflation Factors (VIFs) and Tolerance) between the main effects and the interaction term, and to facilitate interpretation of the regression coefficients. The interaction term (LA × education) was computed within the PROCESS macro. The significance of moderation effects was evaluated by testing the interaction term between LA and education. Conditional effects were probed using the pick-a-point approach implemented in the PROCESS macro for SPSS, which estimates the effect of the predictor at representative values of the moderator derived from the observed distribution of the data (in this case, 5, 8, 10, 13, and 17 years of education). This approach allows the visualization of how the association between leisure activities and outcomes varies across the range of educational attainment present in the sample. Moderation results are reported as regression coefficients (β), standard errors (SE), t statistics, and *p*-values for the main and interaction terms of the regression model, R^2^chng (as a measure of effect size of the interaction terms), while 95% confidence intervals are reported for the predictors and for conditional effects estimated using the pick-a-point procedure. To account for multiple comparisons, only moderation models in which the main effect survived the Bonferroni correction (⍺ set at 0.05/18, *p* = 0.003) were considered statistically significant and subsequently reported in the Results section. Before interpreting the moderation analyses, the assumptions of linear regression were evaluated. Linearity and homoscedasticity were assessed by inspecting scatterplots of standardized residuals against standardized predicted values. In contrast, the normality of residuals was examined through visual inspection of histograms and normal probability (Q-Q) plots of standardized residuals.

## 3 Results

### 3.1 Demographic and clinical characteristics

As shown in Table 1, there was a significant difference in mean age between groups (*F*_3, 282_ = 17.8, *p* < 0.001) due to the fact that AD patients were older than those from any other groups (HS and SCD individuals, *p* < 0.001 in both comparisons; a-MCI patients, *p* = 0.016). In addition, a-MCI patients were older than HS (*p* = 0.016). There was also a significant difference in years of formal education (*F*_3, 282_ = 12.3, *p* < 0.001) across groups. *Post-hoc* analysis revealed that AD and a-MCI patients were less educated than SCD individuals and HS (*p* < 0.001 AD vs. SCD, AD vs. HS and a-MCI vs. HS; *p* = 0.01 a-MCI vs. SCD). Sex distribution differed significantly between AD and a-MCI patients (Chi-square = 6.83, df = 1, *p* = 0.009), in the absence of other between-group differences (AD vs. SCD: Chi-square = 0.54, df = 1, *p* = 0.463; AD vs. HS: Chi-square = 2.12, df = 1, *p* = 0.145; a-MCI vs. SCD = Chi-square = 1.76, df = 1, *p* = 0.184; a-MCI vs. HS: Chi-square = 0.95, df = 1, *p* = 0.328; SCD vs. HS: Chi-square = 0.24, df = 1, *p* = 0.627). Significant differences were observed in MMSE scores (*F*_3, 282_ = 19.2, *p* < 0.001) due to the fact that AD patients showed lower scores than all other groups (*p* < 0.001 in each comparison). As expected, there were significant differences in MTA scores (*F*_3, 282_ = 57.4, *p* < 0.001) with AD patients showing greater atrophy than all other groups (*p* < 0.001 for all comparisons), and patients with a-MCI exhibiting more atrophy than both SCD individuals and HS (*p* < 0.001).

**Table 1:** Demographic and clinical features.

Variables	AD *N* = 82	a-MCI *N* = 98	SCD *N* = 39	HS *N* = 67
Age (mean± SD)	74.6 ± 6.9*§°	71.0 ± 8.1#	67.5 ± 7.9	65.5 ± 8.9
Years of formal education (mean ± SD)	10.2 ± 4.8*°	11.3 ± 4.7#£	14.0 ± 4.1	14.0 ± 4.0
Sex F/M	56/26§	48/50	24/15	38/29
MMSE score (mean± SD)	21.4 ± 4. *§°0	26.8 ± 2.0	27.5 ± 3.7	27.5 ± 4.4
MTA (mean± SD)	2.4 (0.9)* §°	1.9 (0.9) # £	0.6 (0.8)	0.7 (0.9)

*p < 0.05 AD vs. HS; #*p* < 0.05 a-MCI vs. HS; §*p* < 0.05 AD vs. a-MCI;°*p* < 0.05 AD vs. SCD; £*p* < 0.05 a-CI vs. SCD; ^*p* < 0.05 SCD vs. HS.

### 3.2 Neuropsychological assessment

Between group differences were substantially observed across all neuropsychological measures. The expected pattern of neuropsychological results was obtained (see Table 2): AD patients showed significantly lower scores at all tests compared to any other group; a-MCI patients performed worse than SCD and HS groups; finally, no differences were observed between SCD individuals and HS.

**Table 2:** Neuropsychological assessment.

Domain	Test	AD	a-MCI	SCD	HS	*F*_3, 282_	*p*-level
Verbal episodic memory	15-Rey’s words List:						
	Immediate recall (cut-off ≥ 28.5)	27.2 ± 9.7^*§°^	32.8 ± 6.8^#£^	37.4 ± 9.6	43.7 ± 13.4	50.5	<0.001
	Delayed recall (cut-off ≥ 4.6)	2.4 ± 2.6^*°^	5.7 ± 2.7^#£^	6.8 ± 3.2	9.3 ± 4.2	11.8	<0.001
	Hit rates	8.3 ± 3.8^*§°^	11.1 ± 3.4^#£^	11.9 ± 3.1	13.3 ± 3.5	42.0	<0.001
	False Alarms	7.3 ± 6.5^*§°^	4.2 ± 4.5^#£^	2.8 ± 3.7	2.4 ± 4.1	20.8	<0.001
	Short Story test:						
	Immediate recall (cut-off ≥ 3.1)	2.5 ± 2.1^*§°^	4.5 ± 1.7^#£^	5.0 ± 1.9	5.8 ± 2.1	59.1	<0.001
	Delayed recall (cut-off ≥ 2.8)	1.1 ± 1.9^*§°^	4.0 ± 2.2^#£^	4.5 ± 2.2	5.4 ± 2.3	75.8	<0.001
Visuo-spatial episodic memory	Rey’s Complex Figure:						
	Immediate recall (cut-off ≥ 6.4)	5.9 ± 4.9^*§°^	11.1 ± 6.0^#£^	13.6 ± 6.2	15.3 ± 7.3	39.6	<0.001
	Delayed recall (cut-off ≥ 6.3)	5.4 ± 5.3^*§°^	10.8 ± 5.7^#£^	13.2 ± 6.1	14.1 ± 6.9	43.1	<0.001
Verbal short-term memory	Digit Span forward (cut-off ≥ 3.7)	5.3 ± 1.2	5.4 ± 0.9	5.6 ± 1.0	5.7 ± 1.2	0.52	0.669
	Digit Span backward	3.1 ± 1.5^*§°^	3.6 ± 1.0^#£^	3.9 ± 1.2	4.2 ± 1.4	15.9	<0.001
Visuo-spatial short-term memory	Corsi Span forward (cut-off ≥ 3.5)	3.9 ± 1.4	4.5 ± 0.6	4.9 ± 1.1	5.1 ± 1.2	0.38	0.766
	Corsi Span backward	2.9 ± 1.6^*°^	3.8 ± 1.1^#^	4.2 ± 1.3	5.6 ± 6.1	11.1	<0.001
Executive functions	Phonological Word Fluency (cut-off ≥ 17.3)	24.6 ± 8.6^*§°^	30.5 ± 8.7^#£^	33.4 ± 9.7	35.4 ± 12.0	17.8	<0.001
	Modified Card Sorting Test						
	Criteria achieved (cut-off ≥ 4.2)	2.1 ± 1.4^*§°^	3.6 ± 1.9^#£^	4.8 ± 1.8	5.5 ± 1.2	62.0	<0.001
	Perseverative errors	11.6 ± 9.8^*°^	8.9 ± 9.9^#£^	4.6 ± 8.0	2.4 ± 5.4	19.7	<0.001
Language	Naming of objects (cut-off ≥ 22)	24.6 ± 7.6^*°^	28.3 ± 1.8	28.6 ± 3.7	28.1 ± 5.2	3.9	0.010
Reasoning	Raven’s Colored Progressive Matrices (cut-off ≥ 18.9)	24.5 ± 6.3^*°^	26.7 ± 5.3^#£^	28.1 ± 5.5	28.9 ± 6.2	14.9	<0.001
Constructional praxis	Copy of drawings (cut-off ≥ 7.1)	8.1 ± 2.8^*^	9.5 ± 1.6^#^	9.9 ± 1.6	11.9 ± 0.1	6.3	<0.001
	Copy of drawings with landmarks (cut-off ≥ 61.8)	59.9 ± 16.6^*§°^	66.7 ± 3.9^#^	66.6 ± 8.6	65.8 ± 9.9	6.7	<0.001
	Rey’s Complex Figure-Copy (cut-off ≥23.7)	24.3 ± 11.5^°^	29.3 ± 7.4	30.6 ± 6.4	29.4 ± 7.4	4.3	0.006

*p < 0.05 AD vs. HS; #*p* < 0.05 a-MCI vs. HS; §*p* < 0.05 AD vs. a-MCI;°*p* < 0.05 AD vs. SCD; £*p* < 0.05 a-MCI vs. SCD.

### 3.3 Different type of LA’S

#### 3.3.1 Youth

**Figure 1:**
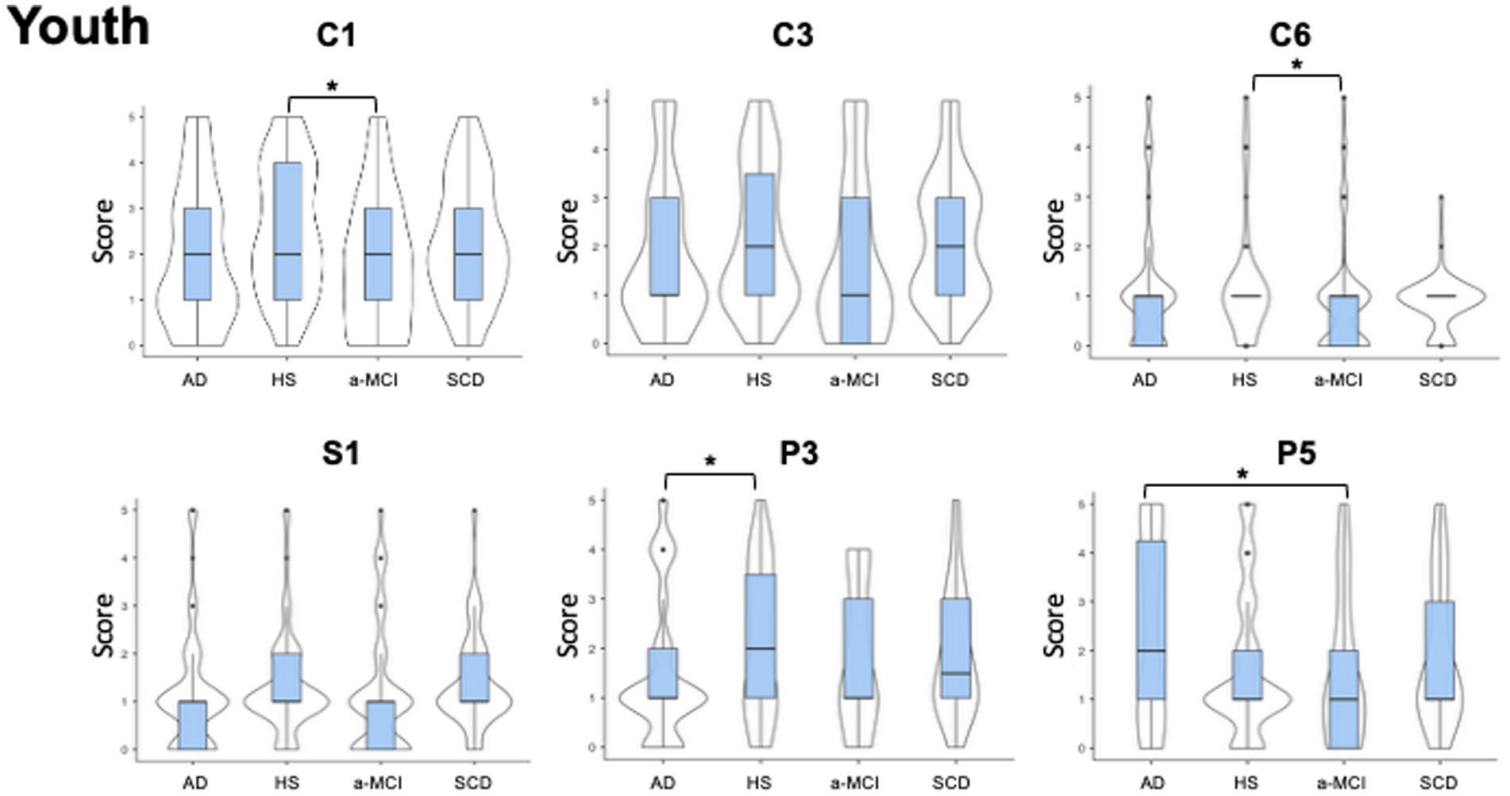
Violin plots of leisure activities pursued during youth. The figure shows the between groups differences in the occurrence of different types of leisure activities performed in the age ranging from 20 to 40 years old. The shape of distribution and the median value (thick line into box plot) are reported. Significant reduction in cognitive (C1 = Participating in lectures/conference/discussion; and C6 = Playing a musical instrument) leisure activities differences were observed between patient with a-MCI and healthy subjects. Patients with AD showed significant decrease in P3 (Practicing individual sports) compared to HS and a significant increase in P5 (Embroidery, sewing, knitting) compared to a-MCsI. See text for further details. AD, Alzheimer’s Disease; a-MCI, amnestic Mild Cognitive Impairment; C1, Participating in lectures/conferences/discussion groups; C3, Producing non-artistic writings; C6, Playing a musical instrument; HS, Healthy Subjects; P3, Practicing individual sports; P5, Embroidery, sewing, knitting; S1, Volunteering; SCD, Subjective Cognitive Decline.

All participants completed the first part of LAs section, assessing activities pursued during youth (age range: 20–40 years) (see Figure 1).

We found significant group differences in the occurrence of cognitive activities, specifically for C1 (*H*_3, 286_ = 10.1, *p* = 0.017) and C6 (H_3,286_ = 8.0, *p* = 0.045). *Post-hoc* analyses revealed that a-MCI patients obtained significantly lower scores than HS in both C1 (a-MCI mean rank = 129.5, HS mean rank = 166.7; W = -4.1, *p* = 0.020) and C6 (a-MCI mean rank = 127.5, HS mean rank = 159.6; W = -3.8, *p* = 0.033). Moreover, significant differences were observed in physical LAs. In particular, for P3 (*H*_3, 286_ = 11.1, *p* = 0.011) AD patients showed lower scores than HS (AD mean rank = 124.6, HS mean rank = 167.3; *W* = −3.1, *p* = 0.009), while they scored higher than a-MCI patients (AD mean rank = 161.7, a-MCI mean rank = 125.6; *W* = 3.1, *p* = 0.009) in P5 (*H*_3, 286_ = 9.2, *p* = 0.027). Finally, we found significant global differences in C3 (*H*_3, 286_ = 7.9, *p* = 0.048) and S1 (*H*_3, 286_ = 8.7, *p* = 0.033); however, *post-hoc* comparisons between pairs of groups did not show significant differences (*p* > 0.05).

#### 3.3.2 Middle age

**Figure 2:**
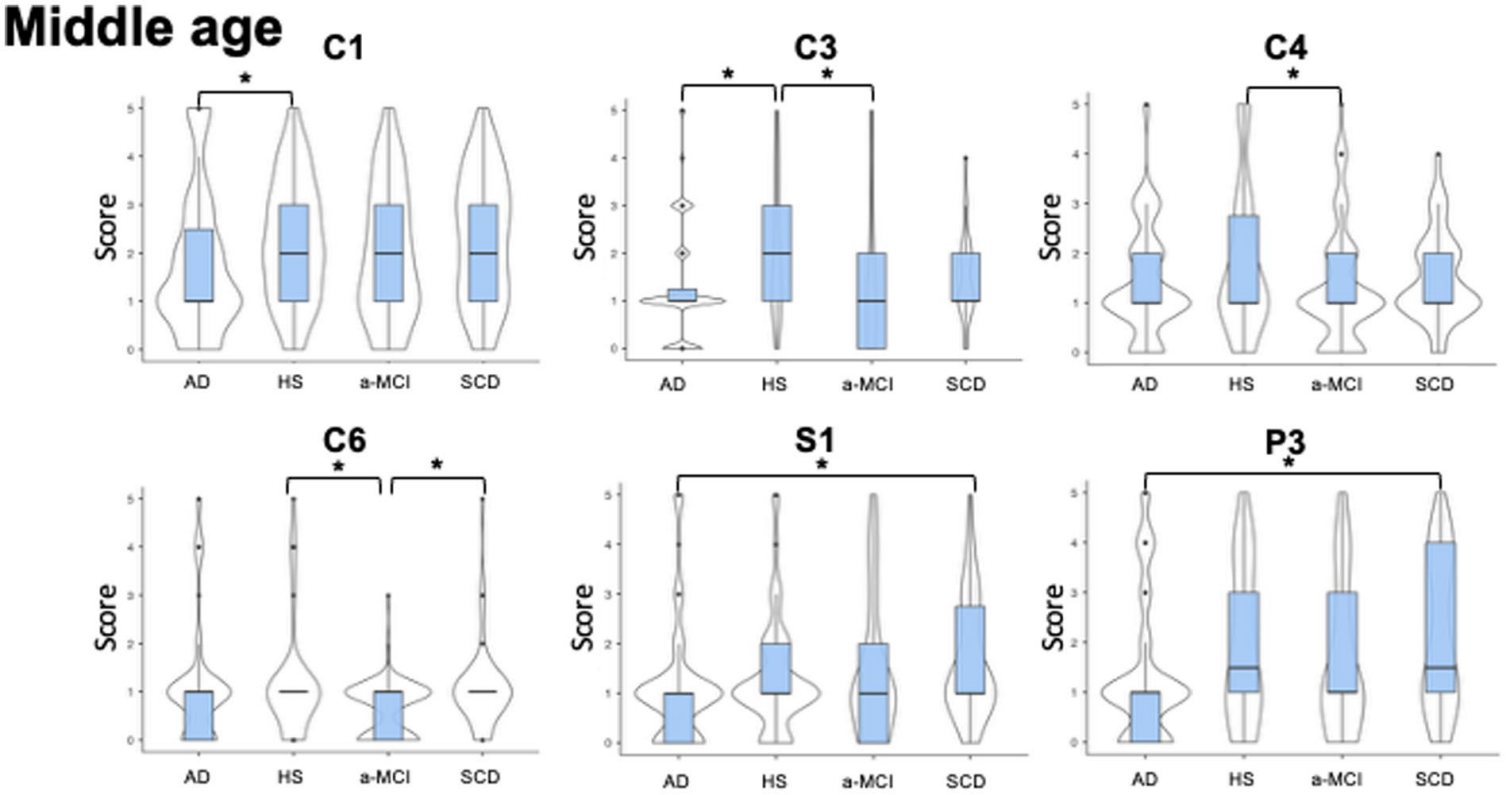
Violin plots of leisure activities pursued during middle age. The figure shows the between groups differences in the occurrence of different types of leisure activities performed in the age ranging from 40 to 65 years old. The shape of distribution and the median value (thick line into box plot) are reported. Significant reduction in cognitive (C3 = Producing non-artistic writing, C4 = Producing artistic works, and C6 = Playing a musical instrument) leisure activities differences were observed between patients with a-MCI and healthy subjects, and also between a-MCI patients and SCD individuals in C6. Patients with AD showed a significant decrease in C1 (Participating in lectures/conferences/discussion groups), C3 (Producing non-artistic writings), compared to HS and significant decrease in S1 (Volunteering) and P3 (Practicing individual sports) compared to SCD. See text for further details. AD, Alzheimer’s Disease; a-MCI, amnestic Mild Cognitive Impairment; C1, Participating in lectures/conferences/discussion groups; C3, Producing non-artistic writings; C4, Producing artistic works; C6, Playing a musical instrument; HS, Healthy Subjects; P3, Practicing individual sports; S1, Volunteering; SCD, Subjective Cognitive Decline.

All participants completed the second part of LAs section of questionnaire (activities pursued during middle age, range: 40–65 years) (see Figure 2).

We found significant differences in the following cognitive activities: C1 (*H*_3, 286_ = 12.7, *p* = 0.005) where AD patients showed lower scores than HS (AD mean rank = 124.4, HS mean rank = 164.0; *W* = −2.9, *p* = 0.020); C3 (*H*_3, 286_ = 14.1, *p* = 0.003) because both AD and a-MCI patients scored lower than HS (AD mean rank = 126.5, a-MCI mean rank = 129.5, HS mean rank = 175.0, *W* = −5.0, *p* = 0.002 and *W* = −3.9, *p* = 0.020, respectively); C4 (*H*_3, 286_ = 9.8, *p* = 0.019), where a-MCI patients obtained lower scores than HS (a-MCI mean rank = 125.4, HS mean rank = 162.5; *W* = −2.8, *p* = 0.027), and finally C6 (*H*_3, 286_ = 11.2, *p* = 0.010) because a-MCI patients scored lower than SCD individuals and HS (a-MCI mean rank = 117.3, SCD mean rank = 162.9, HS mean rank = 161.2; *W* = −4.1, *p* = 0.035 and *W* = −3.8, *p* = 0.020, respectively). Furthermore, significant differences were observed in social and physical activities across groups. Specifically, in S1 (*H*_3, 286_ = 11.1, *p* = 0.011) due to the fact that AD patients showed lower scores than SCD individuals (AD mean rank = 129.5; SCD mean rank = 171.8, *W* = −2.6, *p* = 0.047), and in P3 (H_3, 286_ = 9.7, *p* = 0.021), where AD patients scored lower than SCD individuals (AD mean rank = 123.0; SCD mean rank = 164.6; *W* = −3.9, *p* = 0.029).

#### 3.3.3 Older age

**Figure 3:**
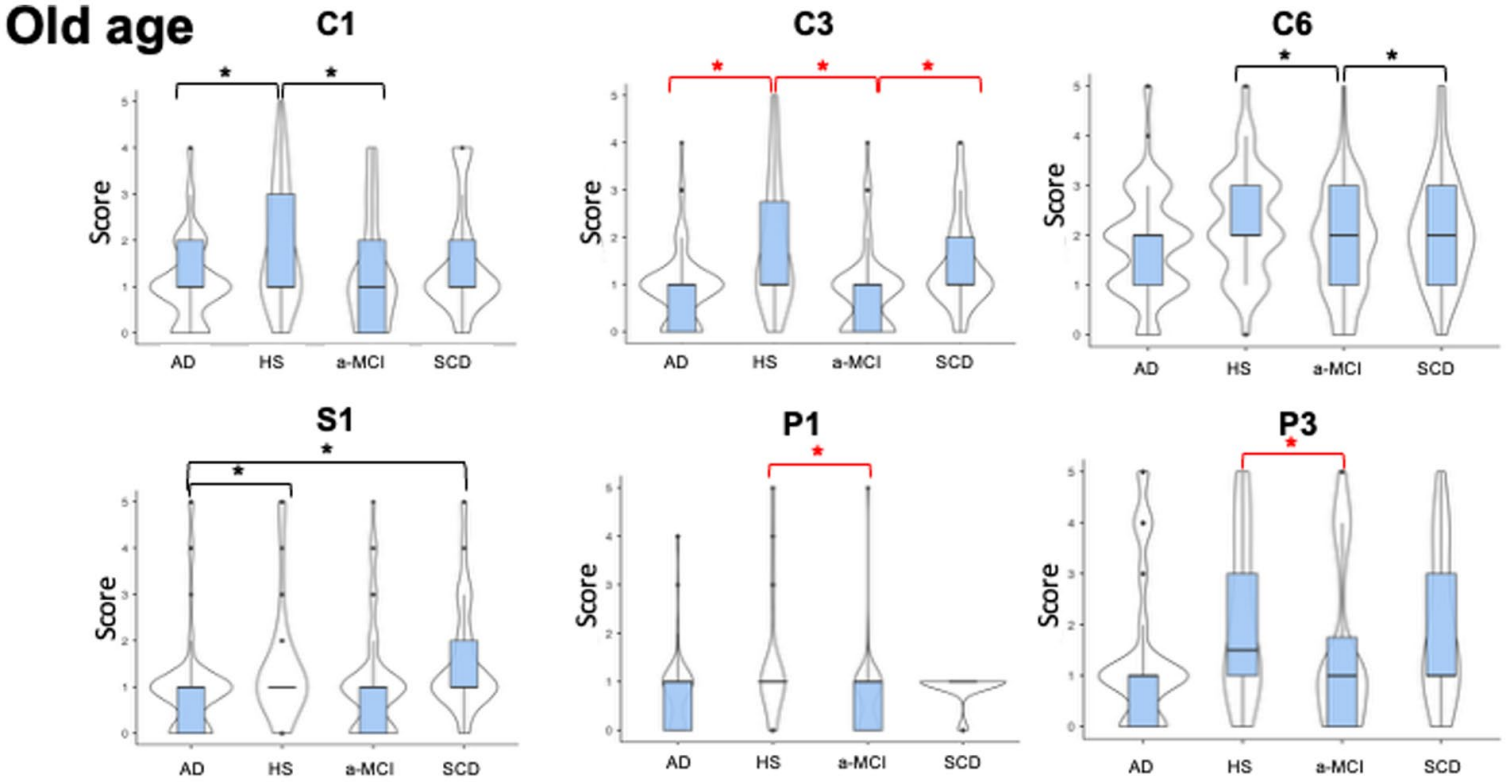
Violin plots of leisure activities pursued during older age. The figure shows the between groups differences in the occurrence of different types of leisure activities performed in the age of 65 years old or older. The shape of distribution and the median value (thick line into box plot) are reported. Significant reduction in cognitive (C1 = Participating in lectures/conferences/discussion groups, C3 = Producing non-artistic writings, and C6 = Playing a musical instrument), (P1 = Playing team sports and P3 = Practicing individual sports) leisure activities differences were observed between patients with a-MCI and healthy subjects, and also between a-MCI patients and SCD individuals in C3 (Producing non-artistic writings) and C6 (Playing a musical instrument). Patients with AD showed significant decrease in C1 (Participating in lectures/conferences/discussion groups), C3 (Producing non-artistic writings), and S1 (Volunteering) compared to HS and a significant decrease in S1 (Volunteering) compared to SCD. See text for further details. AD, Alzheimer’s Disease; a-MCI, amnestic Mild Cognitive Impairment; C1, Participating in lectures/conferences/discussion groups; C3, Producing non-artistic writings; C6, Playing a musical instrument; HS, Healthy Subjects; P1, Playing team sports; P3, Practicing individual sports; S1, Volunteering; SCD, Subjective Cognitive Decline; Red asterisks highlight the results surviving after Bonferroni’s correction.

Two hundred and ten participants (AD = 76; a-MCI = 72; SCD = 24; HS = 38) completed the third part of LAs section (activities pursued during senescence: age range >65) (Figure 3).

For the age of 65 years up to the time of enrolment, we found significant differences in the cognitive LAs, specifically in C1 (*H*_3, 210_ = 9.3, *p* = 0.025), where both patients with AD and a-MCI scored lower than HS (AD mean rank = 98.8; a-MCI mean rank = 95.3, HS mean rank = 133.9; *W* = −3.7, *p* = 0.041 and *W* = −3.5, *p* = 0.050). Similarly, significant differences emerged in C3 (*H*_3, 210_ = 25.4, *p* < 0.001) where AD and a-MCI patients obtained lower scores than HS (AD mean rank = 97.7; a-MCI mean rank = 87.9, HS mean rank = 143.7; *W* = −5.3, *p* < 0.001 and *W* = −5.9, *p* < 0.001, respectively), and a-MCI patients reported lower scores compared to SCD individuals (SCD mean rank = 122.3; *W* = −4.2, *p* = 0.016). Moreover, we observed significant differences in C6 (*H*_3, 210_ = 13.5, *p* = 0.004), where a-MCI patients obtained lower scores than both SCD individuals and HS (a-MCI mean rank = 88.6; SCD mean rank = 115.9, HS mean rank = 132.7; *W* = −3.6, *p* = 0.050 and *W* = −4.7, *p* = 0.005). Significant differences were observed in the first item (S1) of the social activities (see Figure 3) (*H*_3, 210_ = 13.1, *p* = 0.004), where AD patients scored lower than SCD and HS (AD mean rank = 94.9; SCD mean rank = 137.5, HS mean rank = 129.3; *W* = −4.1, *p* = 0.020 and *W* = −3.8, *p* = 0.038). Finally, we found significant differences in P1 (*H*_3, 210_ = 13.7, *p* = 0.003) and P3 (*H*_3, 210_ = 14.3, *p* = 0.002), with a-MCI patients scoring lower than HS in both P1 (a-MCI mean rank = 89.4; HS mean rank = 127.3; *W* = −3.1, *p* = 0.010) and P3 (a-MCI mean rank = 92.1; HS mean rank = 130.3; *W* = −3.1, *p* = 0.010, respectively).

### 3.4 Correlations between education and LAs questionnaire items

#### 3.4.1 Youth

In AD patients, significant positive correlations were found between years of formal education and both C1 (*r* = 0.49, *p* < 0.001) and C2 (*r* = 0.40 *p* < 0.001). In a-MCI patients, education was significantly associated with C1 (*r* = 0.64, *p* < 0.001), C2 (*r* = 0.40, *p* < 0.001), C3 (*r* = 0.48, *p* < 0.001), S1 (*r* = 0.33 *p* = 0.001), S5 (*r* = 0.30, *p* = 0.002) and P1 (*r* = 0.36, *p* < 0.001). Individuals with SCD showed significant associations with C1 (*r* = 0.56, *p* < 0.001), C3 (*r* = 0.54, *p* < 0.001) and C5 (*r* = 0.47, *p* = 0.002). Finally, HS demonstrated significant associations between education and C1 (*r* = 0.67, *p* < 0.001), C2 (*r* = 0.39, *p* = 0.001) and C3 (*r* = 0.61, *p* < 0.001).

#### 3.4.2 Middle age

In AD patients, years of education were significantly correlated with C1 (*r* = 0.38, *p* < 0.001). In a-MCI patients we observed significant correlations between education and C1 (*r* = 0.53, *p* < 0.001), S1 (*r* = 0.30, *p* = 0.003) and P1 (*r* = 0.31, *p* = 0.002). The SCD group showed significant correlations between education and C1 (*r* = 0.61, *p* < 0.001). Finally, HS showed significant associations between education and C1 (*r* = 0.52, *p* < 0.001), C2 (*r* = 0.41, *p* = 0.001) and C3 (*r* = 0.65, *p* < 0.001).

#### 3.4.3 Older age

In a-MCI patients, years of education were significantly correlated with C3 (*r* = 0.44, *p* < 0.001), S1 (*r* = 0.39, *p* = 0.001) and P1 (*r* = 0.35, *p* = 0.003). Additionally, we found significant association in HS group between education and C1 (*r* = 0.50, *p* = 0.001) and C3 (*r* = 0.52, *p* = 0.001).

### 3.5 Relationship between cognition and LAs questionnaire items

#### 3.5.1 Youth

In a-MCI patients, a significant correlation was observed between C1 and MMSE scores (*r* = 0.30, *p* = 0.003), while, in HS, significant correlations were observed between P3 and MMSE scores (*r* = 0.42, *p* = 0.003), P3 and RAVLT-IR scores (*r* = 0.52, *p* < 0.001), P3 and RAVLT-DR scores (*r* = 0.44, *p* = 0.002).

#### 3.5.2 Middle age

In a-MCI patients, P5 was significantly correlated with MMSE scores (*r* = 0.50, *p* < 0.001). In SCD individuals, significant correlations were observed between S2 and RAVLT-DR scores (*r* = 0.52, *p* = 0.001). Finally, in HS, P3 was significantly correlated with MMSE (*r* = 0.50, *p* < 0.001) and RAVLT-IR scores (*r* = 0.46, *p* = 0.001).

#### 3.5.3 Older age

No significant correlations were observed in any group.

### 3.6 Moderation effect of education on the relationship between LAs and cognition

#### 3.6.1 Youth

Only results from moderation models in which the main effect remained significant after Bonferroni correction are reported. Multicollinearity diagnostics indicated no evidence of collinearity among the predictors (all VIFs< 2 and Tolerance< 1), suggesting that multicollinearity did not compromise the model estimates (see Supplementary Table S1 for details). Additionally, the assumptions of linear regression were examined: visual inspection of standardized residuals indicated approximate normality and homoscedasticity, and scatterplots of residuals versus predicted values supported the assumption of linearity.

**Figure 4:**
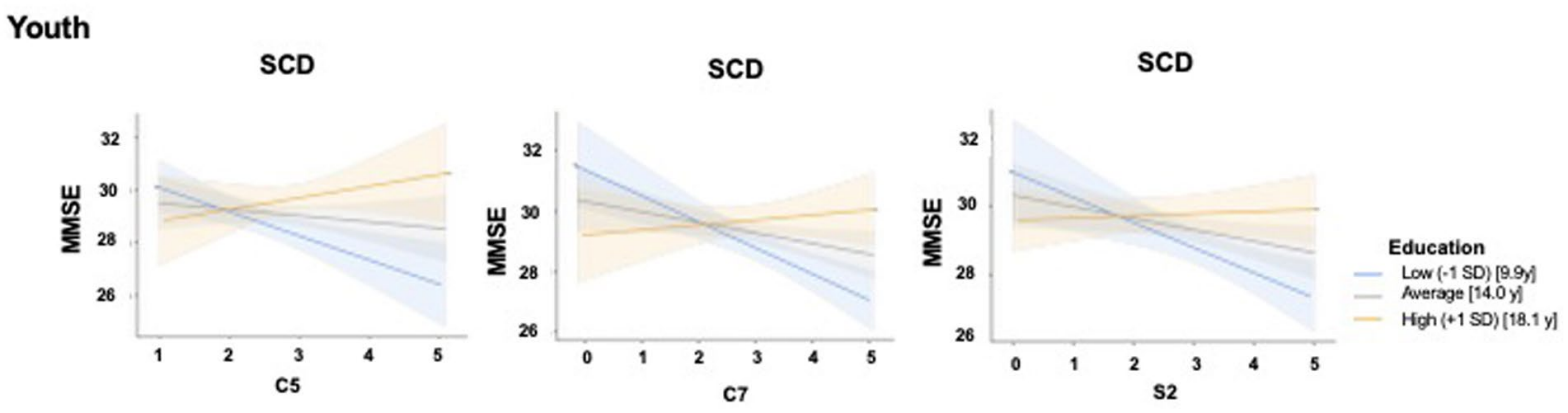
Moderating effect of educational attainment on the relationship between specific leisure activities pursued during youth, and global cognition in individuals with subjective cognitive decline. The figure illustrates the interaction effects of formal education and specific leisure activities pursued during youth on the current global cognition SCD group. The panels illustrate the moderating role of education on the relationship between specific leisure activities as going to the cinema, theater, museums, art galleries (C5) in the left, playing structured games (C7) (in the center), and going to restaurants, bars, or cafes with friends or relatives (S2) (in the right) and the Mini-Mental State Examination (MMSE) scores in individuals with subjective cognitive decline (SCD). X-axis: representing the scores for C5, C7, and S2, respectively; Y-axis: predicted MMSE scores as a proxy for global cognitive function; interaction lines: regression slopes are plotted for three levels of education: low (−1 SD, blue line, corresponding to 9.9 years of education), average (gray line, corresponding to 14.0 years of education), and high (+1 SD, yellow/orange line corresponding to 18.1 years of education). Shaded areas represent 95% confidence intervals. The plots demonstrate a significant interaction where individuals with higher educational attainment (+1 SD) maintain stable or higher global cognitive performance despite variations in specific test scores, compared to those with lower education levels. MMSE, Mini-Mental State Examination; SCD, Subjective Cognitive Decline; SD, Standard Deviation; *y* = years of education.

When considering the activities pursued during youth, significant main effects, were found in a-MCI patients for several cognitive (model including X = C5, Y = MMSE and M = Education: *R* = 0.37, *F*_3, 93_ = 4.95, *p* = 0.003; model including X = C6, Y = MMSE and M = Education: *R* = 0.41, *F*_3, 93_ = 6.28, *p* = 0.003; model including X = C8, Y = MMSE and M = Education: *R* = 0.39, *F*_3, 93_ = 5.52, *p* = 0.001) and physical activities (model including X = P5, Y = MMSE and M = Education: *R* = 0.43, *F*_3, 93_ = 7.25, *p* = 0.002). In the SCD group, we observed in cognitive and social activities significant main effect (model including X = C5, Y = MMSE and M = Education: *R* = 0.60, *F*_3, 34_ = 6.24, *p* = 0.001; model including X = C7, Y = MMSE and M = Education: *R* = 0.64, *F*_3, 34_ = 8.04, *p* = 0.003; model including X = S2, Y = MMSE and M = Education: *R* = 0.65, *F*_3, 34_ = 8.41, *p* = 0.003) and interaction effects (model including C5 × Education: R^2^chng = 0.15, *F*_1, 34_ = 8.05, *p* = 0.007; model including C7 × Education: R^2^chng = 0.16, *F*_1, 34_ = 9.57, *p* = 0.004; model including S2 × Education: R^2^chng = 0.27, *F*_1, 34_ = 16.1, *p* < 0.001). Table 3 reports the beta values of the predictors and the conditional effects of significant interactions. Figure 4 illustrates the interaction effects.

**Table 3:** Moderation analyses results for LAs’ pursued during youth.

Group	Effects of the model				Lower CI-95%	Upper CI-95%	Interaction	Conditional effects of × at different levels of moderator				Lower CI-95%	Upper CI-95%
	Predictors	Beta coefficient	*t*	*p*-values				Levels of moderator	Effect	*t*	*p*-value		
a-MCI	C5	−0.29	−0.52	0.603	−1.39	0.81							
	Education	0.15	1.19	0.235	−0.11	0.42							
	C5xEducation	0.01	−0.06	0.949	−0.09	0.09							
	C6	−0.93	−1.95	0.053	−1.88	0.02							
	Education	0.10	2.03	0.045	0.01	0.21.							
	C6xEducation	0.05	1.30	0.197	−0.03	0.13							
	C8	−0.01	−0.01	0.991	−0.59	0.58							
	Education	0.18	3.04	0.003	0.06	0.30							
	C8xEducation	−0.02	−0.76	0.445	−0.07	0.03							
	P5	−0.29	−0.92	0.358	−0.91	0.33							
	Education	0.14	2.73	0.007	0.04	0.25							
	P5xEducation	−0.01	−0.22	0.825	−0.06	0.05							
SCD	C5	−2.61	−3.34	0.002	−4.20	−1.03	C5xEducation	8	−1.26	−3.49	0.001	−1.99	−0.52
	Education	−0.32	−1.82	0.076	−0.67	0.03		13	−0.41	−1.65	0.111	−0.92	0.09
	C5xEducation	0.17	2.84	0.007	0.05	0.29		17	0.27	0.72	0.473	−0.48	1.01
	C7	−2.25	−3.71	<0.001	−3.48	−1.02	C7xEducation	8	−1.15	−4.11	<0.001	−1.72	−0.58
	Education	−0.32	−2.16	0.038	−0.62	−0.02		13	−0.46	−2.84	0.007	−0.79	−0.13
	C7xEducation	0.14	3.09	0.004	0.05	0.23		17	0.09	0.38	0.703	−0.39	0.57
	S2	−2.55	−4.23	<0.001	−3.78	−1.33	S2xEducation	8	−1.27	−4.19	<0.001	−1.89	−0.66
	Education	−0.40	−2.97	0.005	−0.67	−0.13		13	−0.48	−2.98	0.005	−0.80	−0.15
	S2xEducation	0.16	4.02	<0.001	0.08	0.24		17	0.16	0.96	0.345	−0.18	0.51

#### 3.6.2 Middle age

Only results from moderation models in which the main effect remained significant after Bonferroni correction are reported. Multicollinearity diagnostics indicated no evidence of collinearity among the predictors (all VIFs< 2 and Tolerance< 1), suggesting that multicollinearity did not compromise the model estimates (see Supplementary Table S1 for details). Additionally, the assumptions of linear regression were examined: visual inspection of standardized residuals indicated approximate normality and homoscedasticity, and scatterplots of residuals versus predicted values supported the assumption of linearity.

**Figure 5:**
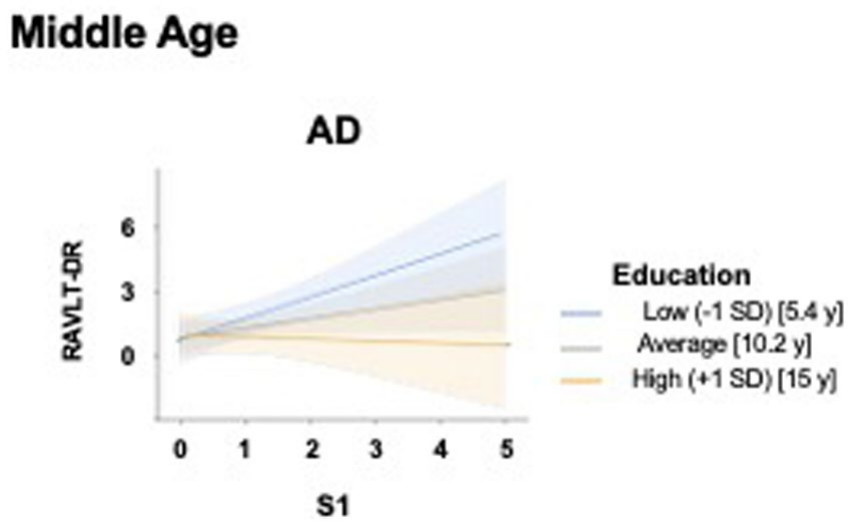
Moderating effect of educational attainment on the relationship between specific leisure activities pursued during middle age, and memory in patients with Alzheimer’s Disease. Interaction effect of formal education and specific leisure activities pursued during middle age on episodic verbal memory in the AD group. The panel illustrates the moderating role of education on the relationship between Volunteering (S1) (x-axis) and Ray Auditory Verbal Learning Test - Delayed Recall (RAVLT-DR) scores (y-axis) in middle-aged individuals with Alzheimer’s Disease (AD). X-axis: Representing the scores for S1; Y-axis: Predicted RAVLT-DR scores as a proxy for memory function; Interaction lines: Regression slopes are plotted for three levels of education: Low (−1 SD, blue line, corresponding to 5.4 years of education), Average (gray line, corresponding to 10.2 years of education), and High (+1 SD, yellow/orange line corresponding to 15 years of education). Shaded areas represent 95% confidence intervals. The plots for this group show that individuals with higher education (+1 SD) exhibit a flatter or slightly negative slope for verbal memory performance as S1 scores increase, whereas those with average and low education maintain a positive association. AD, Alzheimer’s Disease; RAVLT-DR, Ray Auditory Verbal Learning Test - Delayed Recall; SD, Standard Deviation; y = years of education.

As shown in Table 4, in the AD group we observed, for social activities, significant main (model including X = S1, Y = RAVLT-DR and M = Education: *R* = 0.45, *F*_3, 66_ = 5.52, *p* = 0.001) and interaction effects (model including S1 × Education: R^2^chng = 0.10, *F*_1, 66_ = 8.22, *p* = 0.005). Figure 5 illustrates the interaction effect.

**Table 4:** Moderation analyses results for LAs’ pursued during middle age.

Group	Effects of the model				Lower CI-95%	Upper CI-95%	Interaction	Conditional effects of × at different levels of moderator				Lower CI-95%	Upper CI-95%
	Predictors	Beta coefficient	*t*	*p*-values				Levels of moderator	Effect	*t*	*p*-value		
AD	S1	1.92	3.53	<0.001	0.84	3.01	S1xEducation	5	1.20	3.58	<0.001	0.53	1.87
	Education	0.04	0.58	0.562	−0.10	0.19		10	0.48	2.03	0.047	0.01	0.95
	S1xEducation	−0.14	−2.87	0.006	−0.24	−0.04		17	−0.53	−1.23	0.224	−1.40	0.33
a-MCI	C4	−0.41	−0.97	0.334	−1.25	0.43							
	Education	0.14	2.19	0.312	0.01	0.26							
	C4xEducation	0.01	0.19	0.849	−0.07	0.08							
	C6	−0.64	−0.61	0.545	−2.75	1.46							
	Education	0.16	2.68	0.008	0.04	0.28							
	C6xEducation	0.01	0.09	0.922	−0.16	0.18							
	S2	0.77	1.94	0.055	−0.02	1.57							
	Education	0.28	2.92	0.004	0.09	0.47							
	S2xEducation	−0.05	−1.59	0.116	−0.12	0.01							
	S5	0.502	1.37	0.175	−0.23	1.23							
	Education	0.225	3.24	0.002	0.09	0.36							
	S5xEducation	−0.050	−1.47	0.146	−0.11	0.02							
	P5	−0.635	−1.97	0.052	−1.27	0.00							
	Education	0.118	2.27	0.025	0.01	0.22							
	P5xEducation	0.017	0.61	0.544	−0.04	0.07							
SCD	C2	−1.79	−3.72	<0.001	−2.78	0.81	C2xEducation	8	−0.874	−3.93	<0.001	−1.32	−0.42
	Education	−0.27	−2.00	0.052	−0.55	0.00		13	−0.298	−2.32	0.026	−0.56	−0.37
	C2xEducation	0.11	3.24	0.003	0.04	0.18		17	0.162	0.86	0.394	−0.22	0.54
	C5	−1.80	−2.79	0.008	−3.12	−0.49	C5xEducation	8	−0.99	−3.16	0.003	−1.64	0.36
	Education	−0.10	−0.85	0.398	−0.34	0.14		13	−0.49	−2.45	0.019	−0.90	0.08
	C5xEducation	0.10	2.17	0.037	0.01	0.19		17	−0.08	−0.33	0.741	0.63	0.45
	S2	−3.35	−4.96	<0.001	−4.73	−1.98	S2xEducation	8	−1.63	−4.99	<0.001	−2.29	−0.96
	Education	−0.49	−3.57	0.001	−0.78	−0.21		13	−0.55	−3.15	0.003	−0.90	−0.19
	S2xEducation	0.21	4.60	0.001	0.12	0.31		17	0.31	1.42	0.163	−0.13	0.76
	S4	−2.83	−4.02	<0.001	−4.26	−1.40	S4xEducation	8	−1.34	−3.96	<0.001	−2.02	−0.65
	Education	−0.45	−2.85	0.007	−0.76	−0.13		13	−0.40	−2.15	0.038	−0.78	−0.02
	S4xEducation	0.19	3.77	0.001	0.09	0.29		17	0.34	1.41	0.167	−0.15	0.84
	P3	−1.39	−3.30	0.002	−2.24	−0.53	P3xEducation	8	−0.69	−3.52	0.001	−1.08	−0.29
	Education	−0.05	−0.69	0.492	−0.20	0.10		13	−0.25	−2.12	0.042	−0.48	−0.01
	P3xEducation	0.09	2.83	0.008	0.02	0.15		17	0.10	0.61	0.544	−0.24	0.45

In the a-MCI group, significant main effects emerged in the cognitive (model including X = C4, Y = MMSE and M = Education: *R* = 0.40, *F*_3, 91_ = 5.69, *p* = 0.001; model including X = C6, Y = MMSE and M = Education: *R* = 0.38, *F*_3, 91_ = 5.01, *p* = 0.029), social (model including X = S2, Y = MMSE and M = Education: *R* = 0.40, *F*_3, 91_ = 5.56, *p* = 0.001; model including X = S5, Y = MMSE and M = Education: *R* = 0.37, *F*_3, 91_ = 4.82, *p* = 0.003) and physical activities (model including X = P5, Y = MMSE and M = Education: *R* = 0.49, *F*_3, 91_ = 9.67, *p* < 0.001).

**Figure 6:**
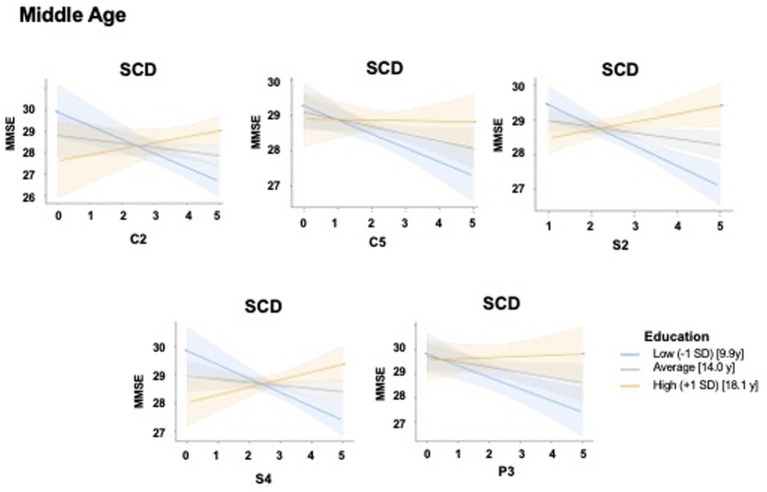
Moderating effect of educational attainment on the relationship between specific leisure activities pursued during middle age and global cognition in individuals with subjective cognitive decline. The figure illustrates the interaction effects of formal education and different leisure activities pursued during middle age on global cognition in the subjective cognitive decline (SCD) group. The panels illustrate the moderating role of education on the relationship between C2 (reading books, newspapers, top left), C5 (going to the cinema, theater, museums, art galleries, top center), S2 (going to restaurants, bars, or cafes with friends or relatives top right), S4 (receiving friends or relatives at home/going to the homes of relatives/friends, bottom left), and P3 (practicing individual sports bottom center) and the Mini-Mental State Examination (MMSE) scores in individuals with (SCD). X-axes: Representing the raw scores for C2, C5, S2, S4, and P3, respectively. Y-axes: Predicted MMSE scores as a proxy for global cognitive function. Interaction lines: Regression slopes are plotted for three distinct levels of formal educational attainment: Low (−1 SD, blue line, corresponding to 9.9 years of education), Average (gray line, corresponding to 14.0 years of education), and High (+1 SD, yellow/orange line corresponding to 18.1 years of education). Shaded areas represent 95% confidence intervals. Similar to the patterns observed in the Figure 4, these plots demonstrate a convergent interaction: individuals with higher education (+1 SD) maintain stable or higher global cognitive performance despite variations in specific leisure activities engagement, while those with average and low education levels show a more pronounced association between increasing leisure activities engagement and declining global cognition. MMSE, Mini-Mental State Examination; SCD, Subjective Cognitive Decline; SD, Standard Deviation; *y* = years of education.

SCD individuals showed, in cognitive, social and physical activities significant main (model including X = C2, Y = MMSE and M = Education: *R* = 0.63, *F*_3, 34_ = 7.43, *p* < 0.001; model including X = C5, Y = MMSE and M = Education: *R* = 0.57, *F*_3, 34_ = 5.43, *p* = 0.003; model including X = S2, Y = MMSE and M = Education: *R* = 0.70, *F*_3, 34_ = 11.15, *p* < 0.001; model including X = S4, Y = MMSE and M = Education: *R* = 0.64, *F*_3, 34_ = 7.76, *p* < 0.001; model including X = P3, Y = MMSE and M = Education: *R* = 0.60, *F*_3, 34_ = 6.26, *p* = 0.002) and interaction effects (model including C2 × Education: R^2^chng = 0.19, *F*_1, 34_ = 10.54, *p* = 0.026; model including C5 × Education: R^2^chng = 0.09, *F*_1, 34_ = 4.71, *p* = 0.037; model including S2 × Education: R^2^chng = 0.31, *F*_1, 34_ = 21.2, *p* = 0.001; model including S4 × Education: R^2^chng = 0.25, *F*_1, 34_ = 7.76, *p* < 0.001; model including P3 × Education: R^2^chng = 0.15, *F*_1, 34_ = 7.99, *p* = 0.008) (Figure 6) illustrates the interaction effects.

#### 3.6.3 Older age

Only results from moderation models in which the main effect remained significant after Bonferroni correction are reported. Multicollinearity diagnostics indicated no evidence of collinearity among the predictors (all VIFs< 2 and Tolerance< 1), suggesting that multicollinearity did not compromise the model estimates (see Supplementary Table S1 for details). Additionally, the assumptions of linear regression were examined: visual inspection of standardized residuals indicated approximate normality and homoscedasticity, and scatterplots of residuals versus predicted values supported the assumption of linearity.

**Figure 7:**
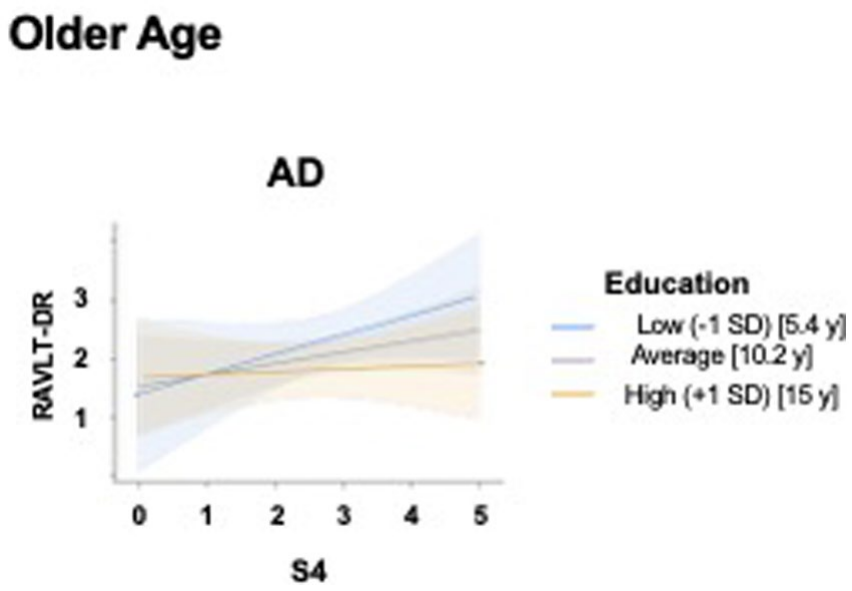
Moderating effect of educational attainment on the relationship between specific leisure activities pursued during older age, and memory in patients with Alzheimer’s Disease. The interaction plot illustrates how formal education levels influence the relationship between the S4 (Receiving friends or relatives at home / Going to the homes of relatives/friends) pursued during older age and Ray Auditory Verbal Learning Test-Delayed Recall (RAVLT-DR) scores in patients with Alzheimer’s Disease. Interaction lines: Regression slopes are displayed for three levels of education: Low (−1 SD, blue line, corresponding to 5.4 years of education), Average (gray line, corresponding to 10.2 years of education), and High (+1 SD, yellow/orange line corresponding to 15 years of education). The shaded regions represent 95% confidence intervals. In AD group, a distinct moderation pattern is observed: while individuals with average and low education levels show a positive association between S4 and RAVLT-DR, those with higher education (+1 SD) demonstrate a remarkably stable (flat) performance across the range of S4 scores. AD, Alzheimer’s Disease; RAVLT-DR, Ray Auditory Verbal Learning Test—Delayed Recall; SD, Standard Deviation, y = years of education.

In the AD group, for Social activities only, significant main (model including X = S4, Y = RAVLT-DR and M = Education: *R* = 0.47, *F*_3, 62_ = 6.04, *p* = 0.001) and interaction effects (model including S4 × Education: R^2^chng = 0.10, *F*_1, 62_ = 78.43, *p* = 0.005) were observed (Table 5, Figure 7) illustrates the interaction effect.

**Table 5:** Moderation analyses results for LAs’ pursued during old age.

Group	Effects of the model				Lower CI-95%	Upper CI-95%	Interaction	Conditional effects of × at different levels of moderator				Lower CI-95%	Upper CI-95%
	Predictors	Beta coefficient	*t*	*p*-values				Levels of moderator	Effect	*t*	*p*-value		
AD	S4	2.11	−3.67	<0.001	0.96	3.26		5	1.45	3.80	<0.001	0.69	2.22
	Education	0.26	1.93	0.058	−0.01	0.52		8	1.06	3.63	<0.001	0.48	1.64
	S4xEducation	−0.13	−2.90	0.005	−0.22	−0.04		17	−0.12	−0.34	0.731	−0.82	0.58

**Figure 8:**
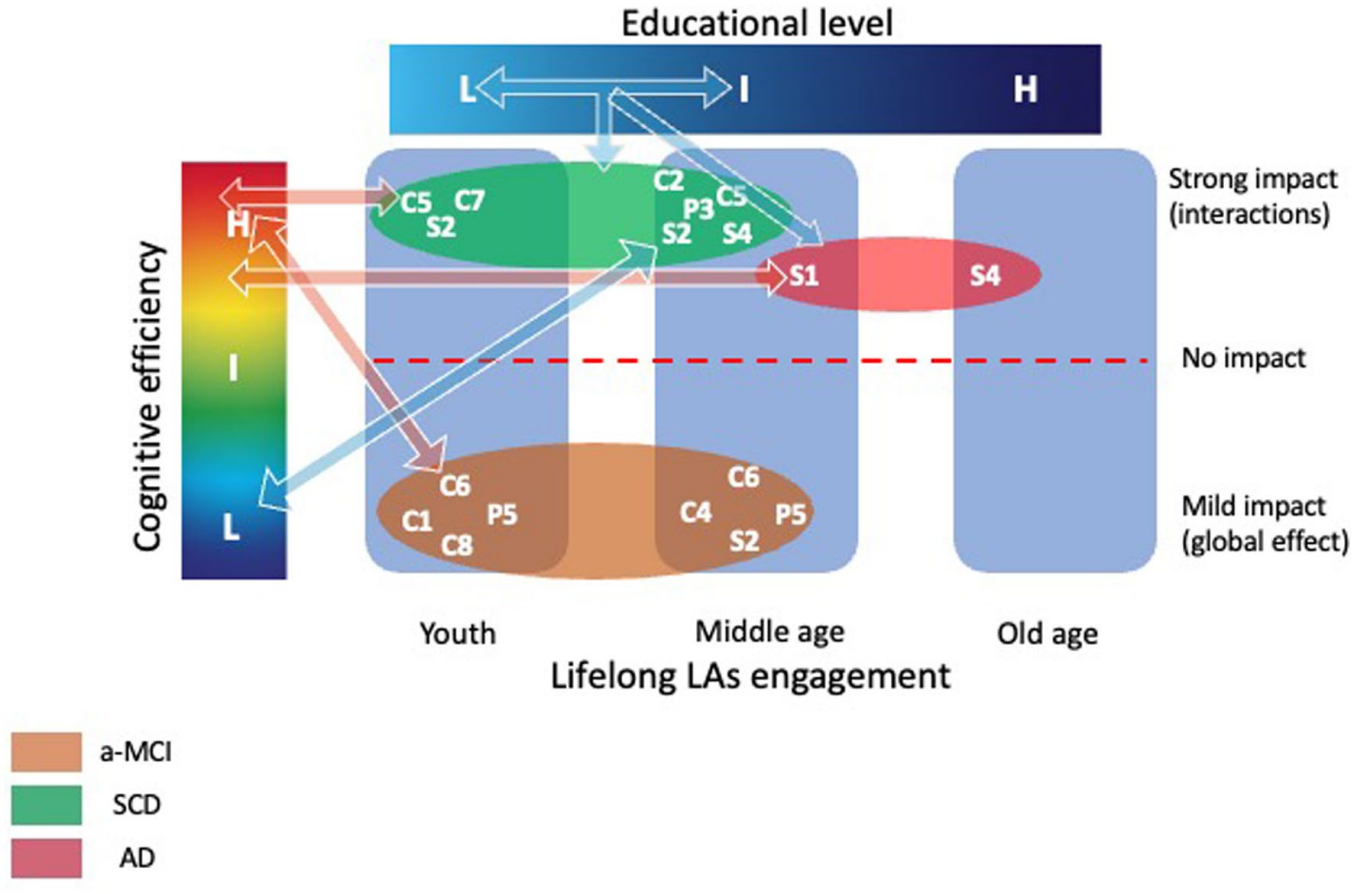
Moderation analyses results. The figure illustrates the main results obtained by the moderation analyses. Strong impact of low and intermediate levels of education was observed on the relationship between cognitive and social activities pursued both during youth (C5 = Going to the cinema, theater, museums, art galleries; C7 = Playing structured games; and S2 = Going to restaurants, bars, or cafes with friends or relatives, positive association) and middle age (C2 = Reading books, newspapers; C5 = Going to the cinema, theater, museums, art galleries; S2 = Going to restaurants, bars, or cafes with friends or relatives; S4 = Receiving friends or relatives at home/Going to the homes of relatives/friends, negative association; and P3 = practice individual sports, negative association) and current cognitive efficiency for SCD individuals. Moreover, in AD group, a strong impact of a lower level of education on the relationship between social activities (S1 = Volunteering and S4 = Receiving friends or relatives at home/Going to the homes of relatives/friends) pursued during middle age and older age on the current cognitive efficiency (positive association) was also observed. Finally, a mild effect due to a general impact of education on the relationship between cognitive, social, and physical activities pursued during youth and middle age on current cognitive efficiency (positive association) was observed for the a-MCI patients. See text for further details. AD, Alzheimer’s Disease; a-MCI, amnestic Mild Cognitive Impairment; C1, Participating in lectures/conferences/discussion groups; C2, Reading books, newspapers; C5, Going to the cinema, theater, museums, art galleries; C6, Playing a musical instrument; C7, Playing structured games; C8, Participating in hobbies; H, High level; I, Intermediate level; L, Low level; LAs, Leisure Activities; P5, Embroidery, sewing, knitting; S1, Volunteering; S2, Going to restaurants, bars, or cafes with friends or relatives; S4, Receiving friends or relatives at home/Going to the homes of relatives/friends; SCD, Subjective Cognitive Decline.

Figure 8 summed the results of moderation analyses.

## 4 Discussion

### 4.1 Main findings

The present study examined patterns of reported engagement in cognitive, social, and physical LAs across different life stages in individuals along the AD continuum, and investigated whether education, considered here as a proxy of cognitive reserve (CR), moderated the association between LAs and current cognitive performance.

Observed group differences in demographic and clinical characteristics align with expected profiles along the AD continuum. AD patients were significantly older than all other groups, including a-MCI, SCD and HS, consistent with disease progression (Hou et al., 2019). Differences in years of formal education, with AD and a-MCI patients being less educated than SCD and HS individuals, have implications for CR. Lower educational attainment has been associated with an increased risk for dementia (Sharp and Gatz, 2011) and may relate to reduced resilience against neuropathological burden. In this study, education was considered as a proxy of CR, assuming that higher levels of CR may modulate the relationship between lifetime engagement in recreational activities and current cognitive functioning.

Differences in sex distribution between the AD and a-MCI groups further support potential sex-related vulnerability in AD (Fernández et al., 2024). More pronounced MTA in AD patients, followed by intermediate atrophy in a-MCI, is consistent with the typical progression of hippocampal and parahippocampal neurodegeneration. The lack of significant atrophy in SCD and HS groups suggests that macroscopic structural brain changes become evident only during the transition from preclinical to clinical stages of the disease. In addition, general cognitive efficiency, measured by MMSE, showed expected patterns, with AD patients performing significantly worse than all other groups. The absence of significant MMSE differences among non-demented groups (a-MCI, SCD, HS) is consistent with the mild or subjective nature of impairment in these conditions. Neuropsychological findings aligned with a gradient of cognitive decline, from widespread deficits in AD patients to intermediate impairment in a-MCI patients, while SCD and HS individuals performed comparably, suggesting subjective complaints in SCD are not yet accompanied by measurable decline.

In examining the occurrence of specific LAs across the lifespan, significant between-group differences emerged, although some effects did not survive after Bonferroni’s correction. However, given the exploratory nature of this study, which aimed to assess whether particular LAs were performed less frequently by patients with AD throughout their lives, we decided to report all results. This decision was motivated by the highly conservative nature of the Bonferroni adjustment, which increases the likelihood of type II errors (false negatives), especially in studies involving related measures and moderate sample sizes. Overall, the results revealed domain- and group-specific differences in reported lifetime activities across diagnostic groups, providing novel insights into the differential contributions of LAs to cognitive reserve and potential resilience-related processes.

Significant differences emerged in cognitive activities performed during youth. Specifically, a-MCI patients reported lower engagement than HS in activities such as attending lectures, conferences or discussion groups (C1), and in playing musical instruments (C6). These findings are consistent with previous evidence associating engagement in intellectually stimulating activities during early adulthood or youth with a higher level of cognitive reserve (Scarmeas et al., 2001; Stern, 2002; Scarmeas and Stern, 2003; Valenzuela and Sachdev, 2006. Fratiglioni and Wang, 2007; Wang et al., 2019; Almeida-Meza et al., 2021; Savarimuthu and Ponniah, 2024). Engagement in such activities is associated with patterns compatible with more efficient and flexible neural networks, potentially reflecting a “cognitive buffer” against cognitive decline. In particular, continuous learning through lessons and discussions has been linked to stronger memory consolidation and hippocampal function (Terranova et al., 2019; Koch et al., 2016), while musical practice may relate to enhanced neural plasticity through rhythmic and synchronization (Toader et al., 2023). Engagement in complex cognitive activities has been associated with improved executive functions (such as planning, inhibitory control, cognitive flexibility), which are considered important for counteracting progression toward AD. Within the physical LAs, AD patients reported lower participation than HS in individual sports (swimming, running, gym or yoga; P3), supporting evidence linking aerobic physical activity during youth and adulthood with preserved cognitive functioning (Serra et al., 2022b; Gelfo et al., 2024). Notably, the higher participation of AD than a-MCI patients in embroidery, knitting, and sewing (P5) may appear counterintuitive. Several studies have associated these fine-motor handicrafts activities with a lower risk of dementia (Geda et al., 2011; Mashinchi et al., 2024; Hansdottir et al., 2022). We hypothesize that when these activities are carried out in prolonged solitude, without meaningful social interaction, the absence of social stimulation may contribute to reducing the potential cognitive benefits derived from fine motor activity. Repetition of these activities without increasing complexity may fail to provide adequate cognitive stimulation and therefore insufficiently activate learning circuits. Indeed, it is well known that neuroplasticity requires challenges, novelty, and progression (Oltmanns et al., 2017). This explanation appears particularly plausible when such activities are practiced in solitude during youth, a developmental phase characterized by heightened synaptic plasticity and network reorganization. In adulthood or senescence, these activities. When these activities are pursued during adulthood or senescence, they probably play a more compensatory role, exploiting the well-established procedural circuits that are reinforced through repetitive activities. Not all LAs showed *post-hoc* significant differences, suggesting that some activities may be less sensitive markers for dementia-related trajectories, consistent with multidomain models of cognitive reserve. Significant group differences also emerged for several activities pursued during middle age, again C1, C6, and P3, but also in producing non artistic writings (such as writing a diary, letters to friends, composing essays) and artistic works (such as painting, writing poetry, sculpt, dancing, songwriting-C3 and C4- respectively) and in volunteering (S1). Both AD and a-MCI patients showed reduced engagement compared to HS and SCD, with a-MCI individuals displaying particularly low participation across several activities. Specific activities such as maintaining a diary or corresponding with friends could be associated with stronger memory traces, which may support retrograde memory resilience, often impaired in AD and MCI patients (Serra et al., 2010; Serra et al., 2022c; Barnabe et al., 2012). In addition, volunteering may be considered a factor promoting cognitive reserve because it engages multiple cognitive functions, including attention, planning, working memory, and mental flexibility, all of which are central components of executive functions. Moreover, the social interaction strengthens social cognition skills, such as empathy, metalizing, and emotional regulation. We speculate that the repeated engagement in such cognitive and social experiences may promote neuroplasticity and formation of new synaptic connections, thereby supporting more efficient brain functioning even in the presence of risk factors. These results align with previous research highlighting midlife as a sensitive window for modifiable risk and resilience factors, where participation in cognitively, physically, and socially stimulating activities is associated with greater cognitive resilience later in life (Chan et al., 2018; Liu and Lachman, 2020; Gavett et al., 2024). For LAs performed during old age, both AD and a-MCI patients reported lower engagement than HS and SCD individuals. Performing artistic works (C3), team sports (volleyball, football, tennis or basket; P1), and individual sports (P3) remained significant even after Bonferroni’s correction, with a-MCI patients being less engaged than HS participants. In addition, the practice of team sports combines the well-known benefits of aerobic physical activity with those of social interaction. In our opinion, this combination may contribute to the stimulating and enriching effects of such activities (Serra et al., 2018) on cognitive and brain function. Overall, the present results extend previous evidence demonstrating that late-life lifestyle engagement can partially buffer the clinical manifestation of neuropathology. However, they also suggest that the potential protective effect of these activities may be less marked once clinical impairment has emerged. The lower scores observed in both AD and a-MCI patients highlight the critical need for early and sustained engagement in stimulating activities for the cognitive functions, as compensatory behaviors initiated after significant cognitive decline are likely to have a limited effect on cognitive efficiency.

### 4.2 CR and education

We observed significant correlations between years of formal education and engagement in specific LA items across youth, middle age, and old age in all studied groups. These findings reinforce the well-established role of education as a key contributor to CR (Barulli and Stern, 2013; Stern et al., 2020; Wilson et al., 2019; Lövdén et al., 2020; Seyedsalehi et al., 2023; Pinto et al., 2024) and suggest that LAs are directly associated with educational levels, and can therefore be considered potential factors promoting reserve. During youth, AD and a-MCI groups showed significant moderate-to-strong correlations between education and cognitive (C1, C2, C3), social (S1, S5) and physical (P1) activities. Individuals with SCD and HS similarly exhibited associations between education and multiple cognitive activities (C1, C3, C5). These results aligned with CR models emphasizing educational attainment as a critical factor for initial reserve building, facilitating early engagement in intellectually and socially complex activities. The stronger correlations in a-MCI and HS groups may indicate a preserved or greater ability to remain actively engaged in stimulating activities in daily life. During middle age, associations between education and cognitive activity (C1) persisted across clinical groups, while a-MCI and HS groups also showed links with social (S1) and physical (P1) activities. Prior studies consistently highlight midlife as a sensitive period during which education promotes the capacity for engagement in diverse and stimulating activities that contribute to neural plasticity and compensatory mechanisms (Vemuri et al., 2014; Chan et al., 2018). The presence of such correlations in SCD and HS groups further may support the notion of midlife as a pivotal phase for reserve amplification, while reduced correlations in AD may reflect disease progression limiting activity participation. When considering the activities pursued during old age, education was significantly correlated with cognitive (C3) and social (S1) activities in a-MCI patients, and with cognitive activities (C1, C3) in HS. This pattern suggests that, despite clinical impairment, education may still promote the maintenance of certain stimulating activities that underpin residual reserve and functional adaptation. However, the fewer significant associations compared to earlier life stages may indicate diminishing opportunity or capacity to sustain LA’s in advanced age and disease severity.

### 4.3 Life-stage specific LAs

We observed significant correlations between LAs pursued during youth and middle age and cognitive measures. In particular, significant associations were found between C1 pursued during youth and MMSE scores in a-MCI patients, as well as between P3 and MMSE, RAVLT-IR, and RAVLT-DR scores in HS. These findings suggest that specific cognitive and physical activities are linked to cognitive performance, consistent with reserve-related mechanisms that may contribute to resilience even before the onset of clinically significant impairment. It should be noted, however, that in the present study, brain resilience was not directly assessed through structural or functional neuroimaging measures. Therefore, the interpretation of these associations in terms of resilience mechanisms should be considered indirect and primarily informed by prior neuroimaging evidence linking cognitive reserve proxies with neural processes supporting resilience to neuropathology.

In HS, the associations between P3 (practicing individual sports) and multiple cognitive measures (reflecting current general cognitive efficiency, learning abilities and episodic memory retrieval) may be indicative of a relationship with reserve-related processes linked to early-life LAs engagement.

When considering the activities pursued during middle age, a-MCI patients exhibited significant associations between P5 and general cognitive efficiency, while SCD individuals revealed relationships between S2 and delayed memory recall performances. In the former case, as mentioned above, the repetition of fine motor activities relying on the activation of procedural memory circuits may help compensate for the initial cognitive decline observed in the early stages of AD. In the latter case, social interactive activities such as going to restaurants or cafè with friends or relatives may stimulate social cognition, decision making, remembering details, conversations, and managing multiple streams of information included those with emotional value, sustaining memory functions, and contributing to neural plasticity.

Overall, these results underscore the relevance of reserve for cognitive outcomes across the trajectory from subjective complaints to MCI. Notably, also in the HS, the consistent association between practicing individual sports (P3) and both general cognitive efficiency and memory functions suggests that specific reserve-related factors, when engaged in youth and in later stages of life, may be associated with greater resilience to cognitive decline.

We also assessed the moderation effect (see Figure 4) exerted by education on the relationship between LAs and cognitive measures. Some effects were mild, as those observed in a-MCI patients, who showed only a main effect of education on the relationship between cognitive (C5, C6 and C8) and physical (P5) activities pursued during youth and middle age on the current global cognitive efficiency. The present findings indicate that engagement in cognitive and physical activities during youth is significantly associated with better cognitive performance during senescence, and that this effect is consistent across multiple activity types, independent of formal education levels, in the early clinical stages of AD. Conversely, significant moderation effects were observed in SCD individuals. In particular, education positively moderates the association between cognitive (C5, C7) and social activities (S2) and current global cognition. However, this moderation effect varied depending on the level of education. At lower and intermediate education levels (8 years and 13 years), the association between LAs and cognition was significant but negative, whereas at higher levels (17 years) the association became positive, even if not significant. This pattern may reflect heterogeneity related to educational background, rather than a direct effect of education on the effectiveness of these activities. Within a cognitive reserve framework, these findings could be interpreted as reflecting differences in how individuals engage with or benefit from cognitively stimulating experiences across the lifespan. However, alternative explanations, such as selection effects, reverse causation, retrospective measurement limitations, or residual confounding, should also be considered.

When considering the activities pursued in middle age, main effects of moderation involving C4, C6, S2, S5, and P5 on general cognitive efficiency were observed in a-MCI patients, suggesting a general influence of education on the association between LAs and cognition. Conversely, significative interactions effects were observed in AD and SCD groups. Specifically, education negatively moderated the relationship between volunteering activities (S1) and memory performances in AD patients: the relationship between S1 and memory performances was positive and more pronounced at lower levels of education (5 and 10 years), whereas it was no longer significant at higher levels of education (17 years). In other words, the association between volunteering during middle age and memory performance differed across levels of education, being more evident in individuals with low or medium education and not significant in those with higher education. This pattern may indicate that the relationship between volunteering and cognitive performance is not uniform across educational strata, possibly reflecting a greater relative relevance of such activity for individuals with fewer educational resources, while in highly educated individuals the association appears less pronounced.

In SCD individuals, education moderated significantly the relationship between cognitive (C2 and C5) and social (S2 and S4) activities and current global cognitive efficiency. At lower and intermediate levels of education (8 or 13 years), the moderation effect was negative, indicating an inverse relationship between engagement in cognitive (books/newspapers read-C2; going to the cinema, theater, museums or art galleries-C5), and social (going to restaurants or café-S2; receiving friends or relatives at home-S4) LAs and current cognitive functioning. At 17 years of education, the associations were positive but not statistically significant. This pattern suggests that, among highly educated individuals, the number of LAs performed during middle age is less clearly related to current cognitive functioning.

Finally, for LAs pursued during old age, both main and interaction effects were observed in AD patients only, particularly involving education, S4, and current memory performance. At all levels of education, the association between S4 and memory was positive, but it reached statistical significance only at lower (5 years) and intermediate (8 years) education levels. This pattern may indicate that, in AD patients, social engagement (meeting friends and relatives) during old age is more closely related to memory performance in individuals with lower educational attainment, whereas in highly educated individuals, memory variability appears less dependent on differences in social activity levels.

### 4.4 Clinical implications

In summary, this study provides evidence that engagement in cognitive, social, and physical lifestyle activities across the lifespan is associated with cognitive performance patterns that are consistent with reserve-related mechanisms along the AD continuum. The observed group differences align with previous findings indicating that lower educational attainment represents a risk factor for dementia and may be linked to reduced resilience (Xu et al., 2016). Reduced participation in intellectually stimulating activities during youth, such as attending discussions (C1) or playing music (C6) in a-MCI patients, alongside significantly lifetime engagement in aerobic individual sports (P3) in AD patients, supports the hypothesis that early engagement in cognitively and physically stimulating activities may be linked to a greater cognitive buffer against neurodegeneration, although causal interpretations cannot be inferred from the present data. Midlife was identified as a critical window, where reduced participation in demanding cognitive and social activities (C3: maintaining a diary; S1: volunteering) in patients’ groups significantly associated with later-life cognitive outcomes, with diary maintenance hypothesized to reinforce memory traces, rendering retrograde memory more resistant to impairment. Formal education consistently reinforced its association with CR, although its moderating role on the *LAs-cognition* relationship appeared complex and non-linear. Significant associations between specific youth LAs and current cognition were observed in a-MCI patients independently of education levels, whereas in AD patients, certain social activities performed in middle or late life (e.g., volunteering or social meetings) were more strongly associated with memory performance among individuals with low or intermediate education. However, this pattern may reflect differences in baseline cognitive resources rather than indicating a uniform relationship between leisure activities and cognition across individuals.

Conversely, poor schooling may attenuate the potential effectiveness of LAs in SCD individuals. Overall, these results suggest that sustained engagement in LAs across the lifespan may be relevant for CR development. However, once clinical impairment emerges, the association between LAs and cognitive efficiency may become more limited. It appears that recreational activities may have a significant impact, particularly in contexts where formal education is limited. It is hypothesized that the consistent and ongoing engagement in cognitively and socially enriching activities during the youth years could help preserve cognitive efficiency in individuals facing disadvantages due to cultural or social factors. Nonetheless, the implementation of such practices during middle age appears to have a less significant effect, particularly in individuals with SCD. The present findings confirm that in the AD patients, the impact of social activities undertaken during middle and old age remains an important factor (Serra et al., 2024). The absence of moderation effects in highly educated SCD individuals or in HS participants does not necessarily indicate the absence of reserve-related processes, but may instead reflect more complex interactions involving additional factors. Indeed, it is possible that the associations between education and these other variables may be non-linear or threshold-dependent, or that the education moderating role emerges only in interaction with such additional factors as demographic variables, genetic factors, brain structural and functional efficiency, and more sensitive cognitive measures. We hypothesize here that years of formal education are not a sensitive CR proxy for individuals highly educated or in heathy aging. The stratification both for years of schooling and different types of educational attainment may be a more sensitive index to capture the CR effect during the successful aging or during the earliest stage of disease.

Further research using non-linear or multivariate approaches may help to clarify the nature of these intricate relationships.

### 4.5 Limitations

The present findings must be interpreted in light of some limitations. First, some complex or counterintuitive interaction patterns that emerged from the moderation analyses, especially within the SCD group, do not necessarily imply that education directly enhances or diminishes the effect of specific LAs. Instead, they may reflect heterogeneous trajectories, compensatory behavioral adaptations, reverse causation (e.g., increased activity engagement following the perceived subjective decline), or unmeasured confounders. Second, the cross-sectional design prevents causal inferences regarding the relationship between LAs, education, and cognitive outcomes. While significant associations were observed, it is not possible to determine whether engagement in LAs contributed to cognitive resilience or if individuals with better cognitive functioning were more likely to engage in these activities. Longitudinal studies are required to clarify directionality. Third, a potential limitation concerns the retrospective reconstruction of LAs. Questions were designed to assess typical and repeated activities rather than isolated autobiographical episodes, an approach considered more robust when collecting retrospective lifestyle information in populations along the AD continuum. Although recall bias cannot be entirely excluded, similar approaches are widely adopted in studies investigating lifetime cognitive reserve proxies (Hultsch et al., 1999; Helzner et al., 2007; Nucci et al., 2012; Garba et al., 2020). In addition, it is remarkable that even structured assessments of autobiographical memory, such as the Autobiographical Interview (Levine, 2004) and the Autobiographical Memory Interview (AMI) (Kopelman, 1994; Berntsen et al., 2022; Serra et al., 2020), are typically administered directly to patients, including those with AD. Caregiver corroboration may only partially mitigate recall bias, as informants may not have reliable knowledge of activities performed during the patient’s early or midlife. Fourth, the sample size may have limited the detection of smaller effects highlighting the need for replication in larger cohorts. Lastly, years of formal education, used as a proxy for CR, may not fully capture its full multidimensional and complex nature, although it has a large application in the CR research framework. Overall, these limitations suggest that the present results should be interpreted as indicative of associations rather than causal effects, emphasizing the need for longitudinal and multimodal approaches to better understand interactions among education, lifestyle, and cognitive resilience across the lifespan.

## 5 Conclusion

These results are consistent with the idea that the cognitive reserve, shaped by formal education and lifelong engagement in complex and stimulating LAs, may remain relevant across decades. Individuals with a richer educational background tended to report greater engagement in leisure activities that were associated with better cognitive performance. Moderation patterns suggest that cognitive reserve is dynamic and cumulative, with education conferring enduring advantages linked to later-life activity engagement. These findings, together, underscore the need for lifelong, education-sensitive strategies to support cognitive reserve and potentially delay the onset and progression of AD. Prevention programs that encourage early and continuous participation in cognitively, socially, and physically stimulating activities, particularly among individuals with lower formal education, may offer meaningful public health benefits by reducing vulnerability to cognitive decline and progression to Alzheimer’s disease.

## Data Availability

The raw data supporting the conclusions of this article will be made available by the authors, without undue reservation.

## Footnotes

1
https://www.ibm.com/it-it/analytics/spss-statistics-software

## Supplementary material

The Supplementary material for this article can be found online at:

https://www.frontiersin.org/articles/10.3389/fnagi.2026.1736047/full#supplementary-material
