# Supplementary material for: Shaping the cognitive reserve: the role of lifelong enrichment and education in the Alzheimer’s disease continuum
Source: Front Aging Neurosci. 2026 May 14;18:1736047. doi: 10.3389/fnagi.2026.1736047 (PMC13216489; doi:10.3389/fnagi.2026.1736047)
Supplement: Supplementary file 1 [file Data_Sheet_1.pdf]

**Supplementary Materials**

As shown in Table S1 there are no evidence of multicollinearity among the tested predictors. Table S1. Multicollinearity Statistics

| | | Predictors | Tollerance | VIF |
|---|---|---|---|---|
| **Youth** | | | | |
| | **a-MCI** | | | |
| | | **C5** | 0.97 | 1.03 |
| | | **Education** | 0.95 | 1.04 |
| | | **C5xEducation** | 0.98 | 1.02 |
| | | **C6** | 0.99 | 1.01 |
| | | **Education** | 0.99 | 1.01 |
| | | **C6xEducation** | 1.0 | 1.00 |
| | | **C8** | 0.97 | 1.02 |
| | | **Education** | 0.98 | 1.01 |
| | | **C8xEducation** | 0.97 | 1.02 |
| | | **P5** | 0.90 | 1.10 |
| | | **Education** | 0.98 | 1.01 |
| | | **P5xEducation** | 0.90 | |
| | **SCD** | | | |
| | | **C5** | 0.78 | 1.28 |
| | | **Education** | 0.88 | 1.13 |
| | | **C5xEducation** | 0.88 | 1.14 |
| | | **C7** | 0.95 | 1.06 |
| | | **Education** | 0.92 | 1.08 |
| | | **C7xEducation** | 0.88 | 1.14 |
| | | **S2** | 0.93 | 1.08 |
| | | **Education** | 0.82 | 1.22 |
| | | **S2xEducation** | 0.83 | 1.20 |
| **Middle age** | | | | |
| | **AD** | | | |
| | | **S1** | 0.98 | 1.02 |
| | | **Education** | 0.99 | 1.01 |
| | | **S1xEducation** | 0.99 | 1.02 |
| | **a-MCI** | | | |
| | | **C4** | 0.92 | 1.08 |
| | | **Education** | 0.99 | 1.01 |
| | | **C4xEducation** | 0.92 | 1.09 |
| | | **C6** | 0.91 | 1.09 |
| | | **Education** | 0.92 | 1.08 |
| | | **C6xEducation** | 0.98 | 1.02 |
| | | **S2** | 0.99 | 1.00 |
| | | **Education S** | 0.99 | 1.00 |
| | | **2xEducation** | 1.00 | 1.00 |
| | | **S5** | 0.94 | 1.07 |
| | | **Education S** | 0.97 | 1.04 |
| | | **5xEducation** | 0.97 | 1.03 |

| | | | | |
|---|---|---|---|---|
| | | **P5** | 0.98 | 1.02 |
| | | **Education** | 0.99 | 1.01 |
| | | **P5xEducation** | 0.97 | 1.03 |
| | **SCD** | | | |
| | | **C2** | 0.90 | 1.10 |
| | | **Education C2** | 0.95 | 1.05 |
| | | **xEducation** | 0.95 | 1.06 |
| | | **C5** | 0.96 | 1.04 |
| | | **Education C5** | 0.98 | 1.01 |
| | | **xEducation** | 0.97 | 1.03 |
| | | **S2** | 0.99 | 1.00 |
| | | **Education S** | 0.61 | 1.64 |
| | | **2xEducation** | 0.61 | 1.64 |
| | | **S4** | 0.99 | 1.01 |
| | | **Education S** | 0.97 | 1.03 |
| | | **4xEducation** | 0.96 | 1.04 |
| | | **P3** | 0.92 | 1.08 |
| | | **Education** | 0.97 | 1.03 |
| | | **P3xEducation** | 0.94 | 1.07 |
| **Older age** | | | | |
| | **AD** | | | |
| | | **S4** | 0.94 | 1.07 |
| | | **Education S** | 0.98 | 1.02 |
| | | **4xEducation** | 0.95 | 1.05 |
